# Interactions between chloroplast and mitochondrial genomes in 11 *Salix* species

**DOI:** 10.3389/fpls.2025.1693183

**Published:** 2025-11-21

**Authors:** Yeseul Kim, Sumin Jeong, Shukherdorj Baasanmunkh, Youngmoon Kim, Hyeok Jae Choi, Inkyu Park

**Affiliations:** Department of Biology, Changwon National University, Changwon, Republic of Korea

**Keywords:** *Salix*, chloroplast genome, mitochondrial genome, comparative analysis, evolutionary relationships, intracellular gene transfer

## Abstract

**Introduction:**

The genus *Salix*, widely distributed across the Northern Hemisphere, is characterized by its dioecious nature and frequent natural hybridization. It has significant ecological and economic value in landscaping, ornamentation purposes, biomass production, and traditional medicine. Understanding its evolutionary dynamics is crucial for effective conservation and sustainable utilization. While hybridization and intracellular gene transfer offer valuable insights into its genetic architecture and evolutionary trajectories, studies examining both chloroplast and mitochondrial genomes remain limited.

**Methods:**

We sequenced and assembled the chloroplast and mitochondrial genomes of male and female individuals from three closely related *Salix* species, namely *S. pierotii*, *S. babylonica*, and *S. pseudolasiogyne*, and incorporated data from eight additional *Salix* species for comparative analysis. Phylogenetic relationships were reconstructed and divergence times were estimated to elucidate the evolutionary history.

**Results:**

The chloroplast genomes ranged from 155,688 to 155,695 bp, and the mitochondrial genomes from 705,072 to 705,179 bp, both showing similar proportions of repetitive sequences. Phylogenetic analyses revealed two main clades corresponding to the *Salix* and *Vetrix* subgenera, with an estimated divergence time of approximately 25–26 MYA. Discrepancies between chloroplast- and mitochondrial-based phylogenies suggest distinct evolutionary histories, and certain mitochondrial genes showed stronger positive selection than chloroplast genes. Homologous fragments between the two organelle genomes indicate intracellular gene transfer events.

**Discussion:**

The observed alternation between *S. pierotii* and *S. pseudolasiogyne* in the mitochondrial phylogeny may indicate potential gene flow or introgressive hybridization, providing further evidence of complex genomic interactions underlying the diversification of *Salix*. Overall, this study underscores the importance of mitochondrial genome analysis in revealing organelle genome evolution and its role in shaping genetic diversity and evolutionary dynamics within *Salix*.

## 1 Introduction

Chloroplasts and mitochondria likely originated from independent prokaryotic organisms that once lived freely. The endosymbiotic theory states that chloroplasts and mitochondria were engulfed by an ancestral eukaryotic cell, and they gradually transferred most of their genetic material to the nucleus of the host cell over time, eventually evolving into organelles with essential roles in eukaryotic cells (Mereschkowsky, 1905; Wallin, 1927; Gray, 1992; Ku et al., 2015). Despite partial gene transfer to the host nucleus, both organelles maintain intricate communication with the nuclear genome and play key roles in cellular metabolism (Chang et al., 2011; Gray, 2012). In angiosperms, chloroplast genomes are generally conserved in size (120–160 kb) and structurally stable, whereas mitochondrial genomes are larger and more variable (200–800 kb) (Palmer, 1983; Wicke et al., 2011; Daniell et al., 2016; Gualberto et al., 2014; Guo et al., 2016). Inheritance of chloroplasts is predominantly maternal, although biparental or paternal inheritance can occur in some species (Hu et al., 2008; Sakamoto and Takami, 2024). Mitochondrial genomes are mostly maternally inherited, with paternal inheritance observed in certain species (Havey, 1997; Havey et al., 1998; Shen et al., 2025).

Gene transfer between the chloroplast, mitochondria, and nuclear genomes is a common phenomenon in plants, playing a pivotal role in the evolution and function of organelles (Smith, 2014; Emamalipour et al., 2020). This intergenomic exchange not only induces structural changes in organelle genomes but also contributes to the increasing size and complexity of plant mitochondrial genomes over evolutionary time (Timmis et al., 2004; Knoop et al., 2010). Notably, gene transfer from both the nucleus and chloroplast to mitochondria occurs more frequently in plants compared to other eukaryotic organisms (Nugent and Palmer, 1991; Lei et al., 2012). As a result, plant mitochondrial genomes exhibit significant structural diversity, which may promote recombination and genomic rearrangements, potentially enhancing the plant’s ability to adapt to fluctuating environmental conditions (Christensen, 2013). These gene transfer events shed light on the evolutionary trajectories of plant organelle genomes. Moreover, chloroplasts and mitochondria perform complementary yet distinct roles within the cell, each adapting to specific functional demands and environmental pressures over time. Given their evolutionary and functional relationships, comparing chloroplast and mitochondrial genomes offers valuable insights into their shared evolutionary histories, gene flow dynamics, and ecological adaptations, thereby advancing our understanding of the evolution of plant organelle genomes.

Organelle genomes serve as important tools for understanding evolutionary history, genetic exchange, and species relationships (Olmstead and Palmer, 1994; Qiu et al., 2010; Tyszka et al., 2023; Yue et al., 2023). The genus *Salix* (ca. 330–500 dioecious shrub and tree species) is broadly distributed across the Northern Hemisphere and serves as model system due to its frequent hybridization and ecological diversity for studying organelle genome evolution (Argus, 1997; Fang et al., 1999; Skvortsov, 1999; Argus et al., 2010; Dickmann and Kuzovkina, 2014). Within this genus, species have traditionally been classified into three subgenera—*Salix*, *Chamaetia*, and *Vetrix* (Skvortsov, 1999), but more recent molecular phylogenomic analyses propose a revised classification with five subgenera (Chen et al., 2025), reflecting its complex evolutionary history. Although chloroplast and mitochondrial genome comparisons are increasingly applied in other plants such as *Artemisia giraldii* (Yue et al., 2022), *Saposhnikovia divaricata* (Ni et al., 2022), and *Ipomoea batatas* (Li G. et al., 2024), integrated organelle genome analyses in *Salix* remain limited.

*Salix* is characterized by frequent natural hybridization, weak reproductive barriers, and both wind- and insect-mediated pollination, contributing to extensive cytonuclear gene flow (Hörandl et al., 2012; Marinček et al., 2023). While the low plastome variation observed in *Salix* is commonly attributed to recent rapid radiation, postglacial range expansion, and persistent hybridization (Wagner et al., 2018, 2020; He et al., 2021a), molecular dating analyses focusing on shrub willow lineages reveal a more intricate evolutionary trajectory, characterized by ancient diversification, incomplete lineage sorting, and geographic fragmentation (Wagner et al., 2021a). Several studies have suggested hybridization and introgression in *Salix*, primarily based on nuclear genomic data (Gramlich et al., 2018; Sanderson et al., 2023). Gene flow between species resulting from hybridization can lead to phylogenetic incongruence. In particular, serial chloroplast capture—where plastid genomes are transferred across species boundaries through repeated hybridization and backcrossing—has been reported in *Salix*, contributing to cytonuclear discordance (Gambhir et al., 2025). The frequent hybridization, complex lineage diversification, and geographic fragmentation observed in *Salix* make it an ideal model for studying the evolutionary dynamics of organelle genomes, particularly in the context of interspecific gene flow. Previous studies have mostly focused on nuclear or combined nuclear and chloroplast data, while comparative analyses of chloroplast and mitochondrial genomes remain limited. Addressing this gap enables investigation into how hybridization and interspecific gene flow shape the evolutionary dynamics of organelle genomes.

In this study, we aimed to investigate the evolutionary relationships of male and female individuals from three closely related *Salix* species by sequencing their chloroplast and mitochondrial genomes. Three species included *Salix pierotii*, *S. babylonica*, and *S. pseudolasiogyne*. To extend the scope of our analysis, we incorporated additional genomic data for 8 other species from the NCBI database, expanding our dataset to include a total of 11 *Salix* species. Our primary objectives were to: (1) compare the organelle genomes of the species and explore their evolutionary relationships, (2) analyze potential gene transfer events between chloroplast and mitochondrial genomes, and (3) investigate whether genomic evidence of genetic admixture could be observed at the organelle level, thereby providing insights into the adaptive processes influencing the evolution of these species. Given the close relatedness, morphological similarity, and ecological overlap among *S. pierotii*, *S. babylonica*, and *S. pseudolasiogyne*, we hypothesized that these species may have experienced historical gene flow or genetic admixture, potentially leaving detectable signatures in their organelle genomes. As organelle genomes evolve independently of the nuclear genome, they provide complementary perspectives on evolutionary relationships. To test this hypothesis and explore the evolutionary dynamics of their organelle genomes, we sequenced the chloroplast and mitochondrial genomes of both male and female individuals from these species.

## 2 Materials and methods

### 2.1 Sample collection, DNA extraction and sequencing

Male and female individuals of *Salix pierotii* (35°16’04.4”N 128°16’34.7”E), *S. babylonica* (35°10’17.2”N 128°58’27.5”E), and *S. pseudolasiogyne* (35°14’26.1”N 128°41’51.4”E) were separately collected in South Korea. Corresponding voucher specimens were deposited in the Herbarium of Changwon National University (CWNU), Korea, a publicly accessible herbarium. Voucher numbers—NG23042701, NG23042702, SN23050401, SN23050402, CC23050101, and CC23050102—along with detailed collection information are listed in **Supplementary Table 1**. Species identification was initially conducted by Youngmoon Kim, a PhD student at Changwon National University whose research focuses on the taxonomy of the genus *Salix*. The identifications were subsequently verified by Dr. Shukherdorj Baasanmunkh and Professor Hyeok Jae Choi, both plant taxonomists affiliated with the same institution. Total genomic DNA was isolated from fresh leaves following a modified cetyltrimethylammonium bromide method (Allen et al., 2006). DNA purity and concentration were evaluated using a BioDrop uLite spectrophotometer (Biochrom Ltd., Cambridge, UK). Library preparation was carried out with the TruSeq DNA Nano kit (Illumina, San Diego, CA), and sequencing was conducted on the Illumina NovaSeq 6000 platform (**Supplementary Table 2**). Raw reads were subjected to quality control and trimming using FastQC v.0.11.5 and Trimmomatic v.0.36 (Bolger et al., 2014), respectively, with sequencing data summary presented in **Supplementary Table 3**.

### 2.2 Organelle genome assembly and annotation

Clean Illumina paired-end reads were assembled *de novo* into the chloroplast genome using Velvet v.1.2.08 (Zerbino and Birney, 2008), with subsequent validation and refinement via self-mapping in Geneious Prime v.2024.0 (Kearse et al., 2012). Sequencing coverage was confirmed by calculating read depth with Sequence Alignment/Map tools (Li et al., 2009; **Supplementary Figure S1**; **Supplementary Table 4**). Species similarities were identified by performing BLAST analysis (Altschul et al., 1990). The positions of protein-coding sequences, tRNAs, and rRNAs were mapped, and their functions were annotated using PGA v.2019 (Qu et al., 2019), which facilitated the rapid annotation of the chloroplast genome. The mitochondrial genome was assembled by mapping reads to the *Salix wilsonii* genome as reference (NC_064688.1; Han et al., 2022) using the ‘Map to Reference’ tool in Geneious Prime v.2024.0 (Kearse et al., 2012). Completeness and coverage were additionally evaluated through self-mapping (**Supplementary Figure S2**; **Supplementary Table 5**). Gene annotation was carried out using GeSeq (Tillich et al., 2017), and the results were subsequently verified using BLASTn (Chen et al., 2015; **Supplementary Table 6**). All annotations were manually verified and corrected in Geneious Prime v.2024.0 (Kearse et al., 2012). The map was visualized using OGDRAW (Greiner et al., 2019).

### 2.3 Repetitive sequence detection in organelle genome

To detect simple sequence repeats (SSRs) in chloroplasts and mitochondrial genomes, we employed MISA (Beier et al., 2017). For chloroplast genome analysis, the parameters ‘1-10, 2-5, 3-4, 4-4, 5-3, 6-3’ were applied, while ‘1-8, 2-4, 3-4, 4-3, 5-3, 6-3’ were used for mitochondrial genomes, targeting mono- to hexanucleotide motifs. Tandem repeats were detected using Tandem Repeats Finder (Benson, 1999), with a match score of 2, mismatch and indel penalties set to 7, and a minimum alignment score threshold of 50. Additionally, the maximum period size was set to 500, the maximum repeat array size to 2,000,000 bp, and only repeats with at least 90% sequence identity were reported. For the identification of dispersed repeats, REPuter (Kurtz et al., 2001) was employed with a minimum repeat length of 30 bp and a Hamming distance threshold of 3, thereby ensuring selection of significant and sufficiently long repeats. Only repeats with at least 90% sequence identity were selected and confirmed to ensure high confidence.

### 2.4 Phylogenetic tree inference

Sequence data for eight *Salix* species, whose complete chloroplast and mitochondrial genomes are publicly accessible through the NCBI database (https://www.ncbi.nlm.nih.gov/genome/browse/; last accessed March 2024), were retrieved. *Populus davidiana*, a species closely related within the Salicaceae family, served as the outgroup; its complete chloroplast (NC_032717.1) and mitochondrial (NC_035157.1) genomes were also obtained from NCBI (**Supplementary Table 7**). Common protein-coding genes in the 11 *Salix* species were identified using Geneious Prime v.2024.0 (Kearse et al., 2012), and the sequences were then aligned using MAFFT (Katoh et al., 2002). Conserved segments of the alignments were extracted using Gblocks v.0.91b with default settings (Castresana, 2000). For phylogenetic analysis, the optimal substitution models for both chloroplast and mitochondrial genomes were determined using jModelTest v.2.1.10 (Darriba et al., 2012; **Supplementary Table 8**). Bootstrap support values were estimated with 1,000 replicates in MEGA 11 (Tamura et al., 2021), and a Maximum Likelihood (ML) phylogeny was constructed accordingly. Bayesian Inference (BI) analysis was performed using Geneious Prime v.2024.0 (Kearse et al., 2012) over 5,000,000 generations, sampling every 5,000 generations to assess convergence and ensure robustness. The initial 25% of sampled generations were excluded as burn-in to reduce bias in the final tree.

### 2.5 Divergence time estimation

Divergence times among the 11 *Salix* species were estimated using BEAST v.2.7.6 (Bouckaert et al., 2019), based on the shared protein-coding genes. The analysis incorporated 76 chloroplast genes and 28 mitochondrial genes. A GTR substitution model with four rate categories was implemented. A relaxed molecular clock model was applied, and speciation was modeled under a Yule speciation prior. To calibrate the divergence times, two key fossil-based calibration points were utilized: (1) The divergence between *Salix* and *Populus*, with a normal prior distribution having a mean of 48 million years ago (MYA) and a standard deviation of 0.3. This estimate is based on fossil evidence, including *Populus tidwellii* from the early Eocene (Manchester et al., 2006), and reflects a narrow uncertainty range to account for minor variations in previous studies (Wu et al., 2015). (2) The divergence between the subgenera *Salix* and *Vetrix*, constrained within 23.76–33.99 MYA (Wu et al., 2015). Markov Chain Monte Carlo (MCMC) analysis was conducted for 30 million generations, with convergence and sampling adequacy assessed via effective sample size (ESS) values using Tracer v.1.7.2 (Rambaut et al., 2018). The initial 10% of generations were discarded as burn-in, after which maximum clade credibility trees were constructed using TreeAnnotator v.2.7.6 (Bouckaert et al., 2019). Final trees were visualized with FigTree v.1.4 (Rambaut, 2014).

### 2.6 Selective pressure analysis

Selective pressure on protein-coding sequences in 11 *Salix* species was assessed by calculating dN/dS ratio (ω = dN/dS) for both chloroplast and mitochondrial genes, using *Populus davidiana* (NC_032717.1 for chloroplast; NC_035157.1 for mitochondrion) as the reference. The analysis focused on shared protein-coding genes across these species. The ω was calculated using the count-based method yn00 from the PAML package v.4.9 (Yang, 2007), applying the F3X4 codon model. In this context, values greater than one imply positive selection, values close to one denote neutral evolution, and values below one indicate purifying selection. For this purpose, nonsynonymous (dN) and synonymous (dS) substitution rates were calculated, with dN representing amino acid–altering mutations and dS representing silent mutations. To ensure accuracy, undefined (0/0) and infinite (x/0) values were excluded from the analysis.

### 2.7 Chloroplast and mitochondrial genome sequence similarity analysis

To identify gene transfer events, we compared chloroplast and mitochondrial genome sequences of the 11 *Salix* species using BLASTn (Chen et al., 2015). For this analysis, the following parameters were selected: similarity threshold of ≥ 80%, an e-value of ≤ 1e-5, and a minimum sequence length of 50 bp. Gene transfer events identified through this process were visualized using Circoletto (Darzentas, 2010), which generated circular diagrams of sequence alignments, providing a comprehensive representation of transfer events between the two genomes.

## 3 Results

### 3.1 Features of organelle genomes in three *Salix* species

**Figure 1:**
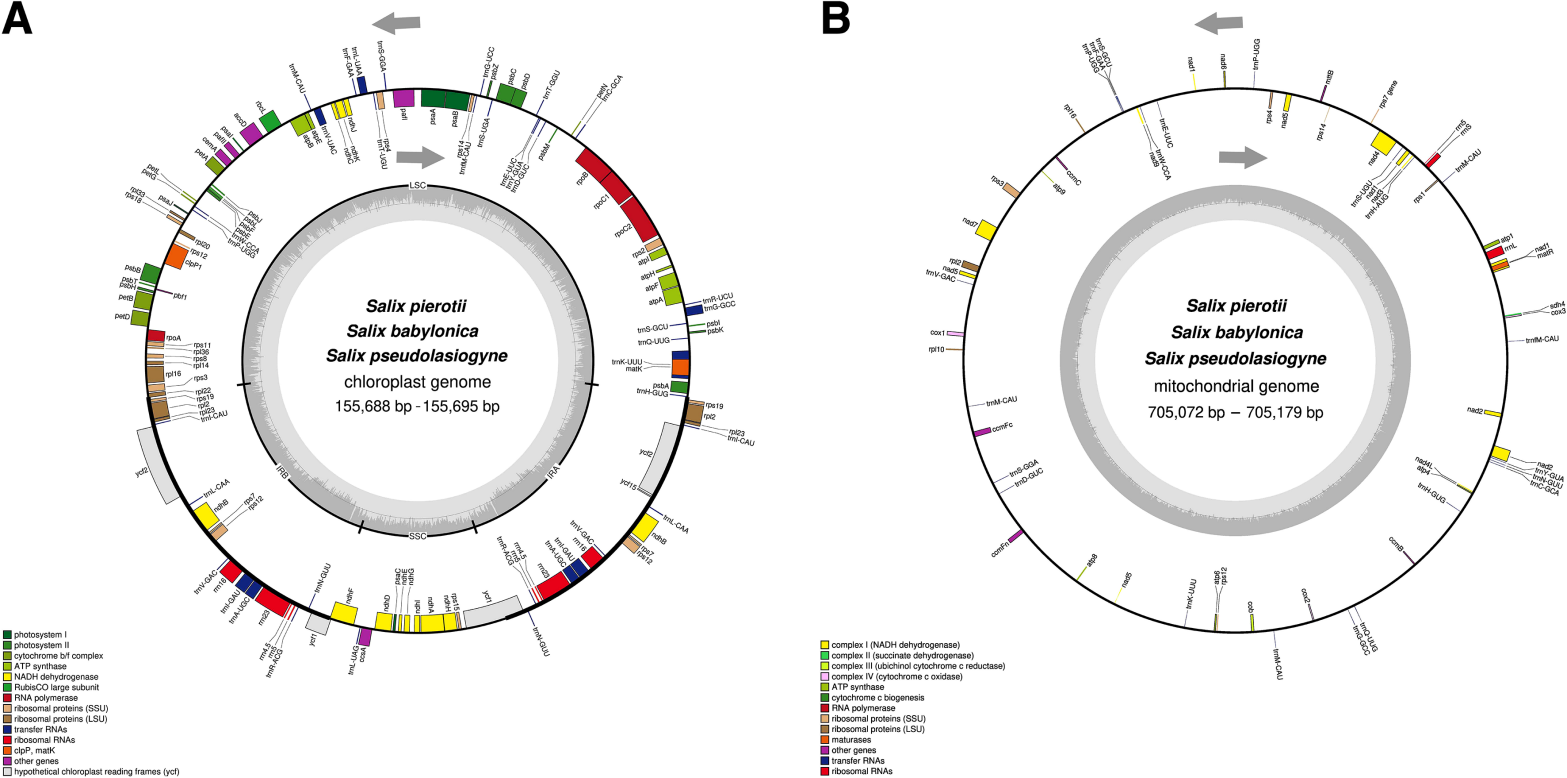
Circular maps of organelle genomes in three *Salix* species. **(A)** Circular map of the chloroplast genome. **(B)** Circular map of the mitochondrial genome. Genes inside the circle are transcribed in the clockwise direction, whereas genes outside the circle are transcribed counterclockwise. Genes are color-coded based on their functions, and the gray area inside the circle represents the GC content.

The complete chloroplast genomes of the three *Salix* species, *namely S. pierotii, S. babylonica, and S. pseudolasiogyne*, ranged from 155,688 to 155,695 bp in length, and displayed the typical quadripartite structure characteristic of chloroplast genomes. The quadripartite structure included four sections: a large single-copy (LSC), a small single-copy (SSC), and two inverted repeat (IRA and IRB) regions (**Figure 1A**; **Supplementary Figure 3**; **Table 1**). The GC content in the chloroplast genomes were approximately 36%, and a total of 111 genes were identified, including 77 protein-coding genes, 30 tRNAs, and 4 rRNAs. Among these, introns were present in 17 genes, with two genes (*pafI* and *clpP1*) each containing two introns (**Supplementary Tables 9**, **10**). The mitochondrial genomes of the three *Salix* species were circular and ranged in size between 705,072 and 705,179 bp, with a GC content of around 44% (**Figure 1B**; **Supplementary Figure 4**; **Table 2**). Each mitochondrial genome of the three *Salix* species comprised 59 genes, including 34 protein-coding genes, 22 tRNA genes, and 3 rRNA genes. Of these, 8 contained introns, and among the tRNA genes, only two (*trnM*-*CAU* and *trnP*-*UGG*) had multiple copies (**Supplementary Table 11**). The mitochondrial genomes were significantly larger—approximately 4.5 times larger—than the chloroplast genomes. In addition, there is a total difference of 52 genes between the two genomes, primarily due to differences in protein-coding genes. Chloroplast genomes contain 43 more protein-coding genes, 8 tRNA genes, and 1 rRNA gene compared to mitochondrial genomes.

**Table 1:** Features of the chloroplast genomes of three *Salix* species (including both male and female individuals).

Species	*S. pierotii*	*S. babylonica*	*S. pseudolasiogyne*
Accession number	PQ842549-50	PQ842551-52	PQ842553-54
Total chloroplast genome size (bp)	155,688	155,695	155,689
Large single copy (LSC) (bp)	84,479	84,469	84,479
Inverted repeat (IR) (bp)	27,446	27,453	27,446
Small single copy (SSC) (bp)	16,317	16,320	16,318
Total number of genes (unique)	111	111	111
Protein-coding gene (unique)	77	77	77
tRNA (unique)	30	30	30
rRNA (unique)	4	4	4
GC content (%)	36.64%	36.63%	36.64%
LSC (%)	34.40%	34.40%	34.40%
IR (%)	41.70%	41.70%	41.70%
SSC (%)	31.00%	31.00%	31.00%

**Table 2:** Features of the mitochondrial genomes of three *Salix* species (including both male and female individuals).

Species	*S. pierotii*	*S. babylonica*	*S. pseudolasiogyne*
Accession number	PQ873106-7	PQ873108-9	PQ873110-11
Total mitochondrial genome size (bp)	705,081	705,179	705,072
Total number of genes (unique)	59	59	59
Protein-coding gene (unique)	34	34	34
tRNA (unique)	22	22	22
rRNA (unique)	3	3	3
GC content (%)	44.80%	44.80%	44.80%

### 3.2 Repetitive sequences in the organelle genomes

**Figure 2:**
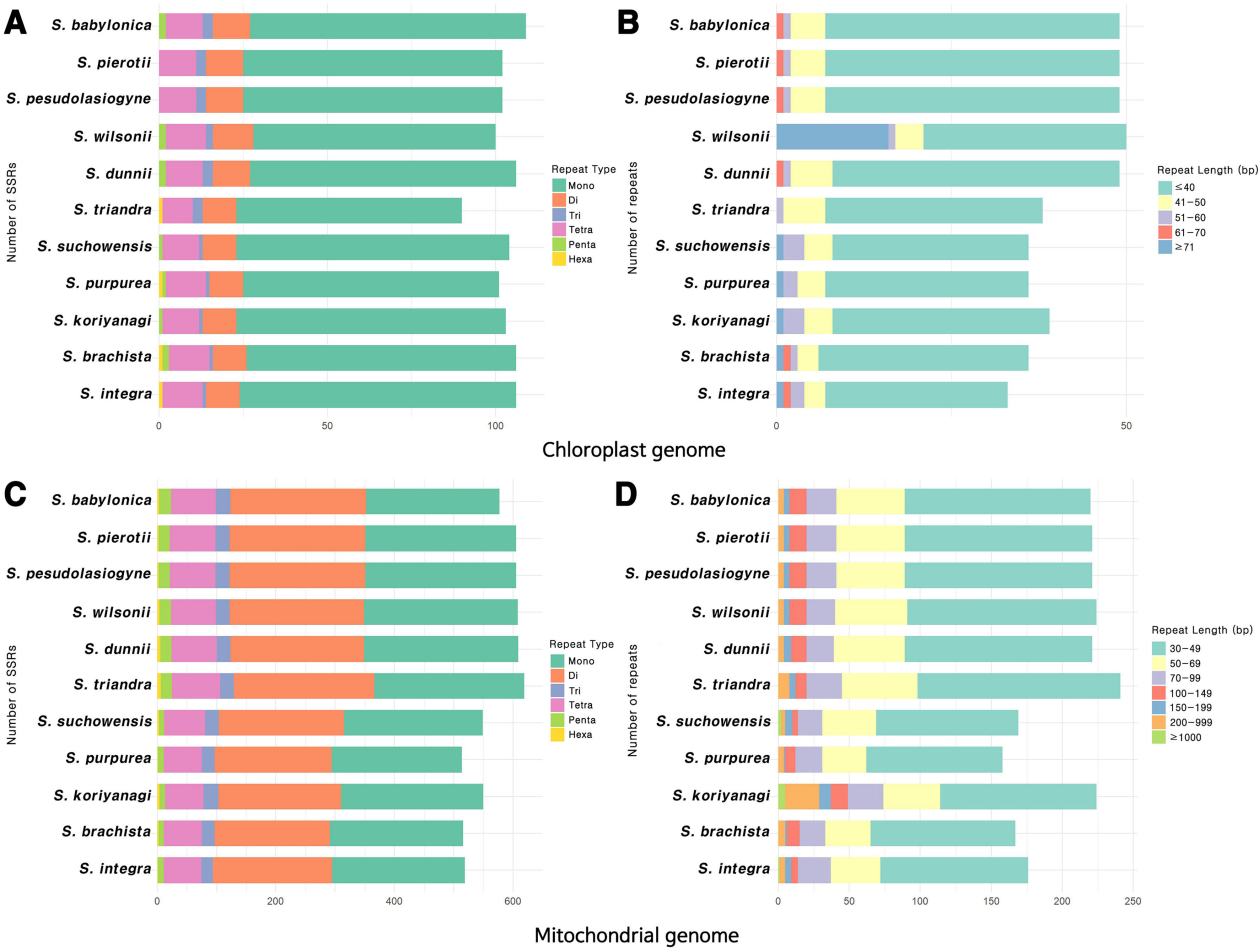
Distribution of repeat sequences in the organelle genomes of the 11 *Salix* species. **(A)** Types of SSR motifs identified in the chloroplast genome. **(B)** Frequency distribution of various repeat lengths in the chloroplast genome. **(C)** Types of SSR motifs identified in the mitochondrial genome. **(D)** Frequency distribution of various repeat lengths in the mitochondrial genome.

The most abundant SSRs in the chloroplast genome were mononucleotide repeats, with A/T repeats being the predominant type (**Figure 2A**; **Supplementary Figure 5A**). Among the dispersed repeats, forward repeats were the most abundant, followed by palindromic repeats, with most of these repeats being < 40 bp in length (**Figure 2B**; **Supplementary Figure 5B**). Tandem repeats primarily ranged from 30 to 51 bp in length, with the highest frequency observed in the 31–40 bp range (**Supplementary Figure 5C**). On the other hand, the mitochondrial genome had a greater abundance of SSRs, predominantly in the form of mononucleotide and dinucleotide repeats, with A/T and AG/CT repeats being especially prevalent (**Figure 2C**; **Supplementary Figure 6A**). The length of dispersed repeats ranged from 30 bp to over 1000 bp, with most falling within the 30–49 bp range (**Figure 2D**). Only forward and palindromic repeats were identified, and tandem repeats ranged from 30 to 91 bp (**Supplementary Figures 6B, C**). The comparison of the chloroplast and mitochondrial genomes across 11 *Salix* species revealed that repeats in the mitochondrial genome were approximately 3 to 8 times longer in size and 4 to 5 times more abundant than those in the chloroplast genome (values have been rounded for simplicity). On average, repetitive sequences accounted for approximately 3% of the total length of the chloroplast and mitochondrial genomes (**Supplementary Figure 7**).

### 3.3 Phylogenetic relationships and divergence estimation of the 11 *Salix* species

Both the chloroplast- and mitochondrial-based trees revealed two major groups, namely the *Salix* subgenus and *Vetrix* subgenus. Interestingly, *S. triandra*, though classified within the *Salix* subgenus, was more closely related to the *Vetrix* subgenus (**Supplementary Figures 8**, **9**). The chloroplast-based tree showed consistent phylogenetic structure in both ML and BI analyses, whereas the mitochondrial-based tree exhibited topological inconsistencies between the two methods. Additionally, topological differences were observed between the chloroplast and mitochondrial trees. Specifically, the chloroplast-based tree identified a monophyletic group within the *Salix* subgenus, which comprised *S. pierotii*, *S. babylonica*, and *S. pseudolasiogyne*. *Salix wilsonii* and *S. dunnii* were also grouped together, and the five species from the *Vetrix* and *Chamaetia* subgenera formed a monophyletic clade. Additionally, *S. suchowensis* was closely related with *S. koriyanagi*, whereas *S. purpurea* showed close relationship with *S. integra*. In contrast, the mitochondrial-based trees showed some discrepancies. The ML and BI trees did not group *S. wilsonii* and *S. dunnii* together. Although the five species from the *Vetrix* and *Chamaetia* subgenera formed a monophyletic clade, the ML tree did not group *S. purpurea* and *S. integra* together. Instead, *S. suchowensis* and *S. koriyanagi* showed a closer phylogenetic relationship. However, in the BI tree, *S. suchowensis* and *S. purpurea* were grouped together.

**Figure 3:**
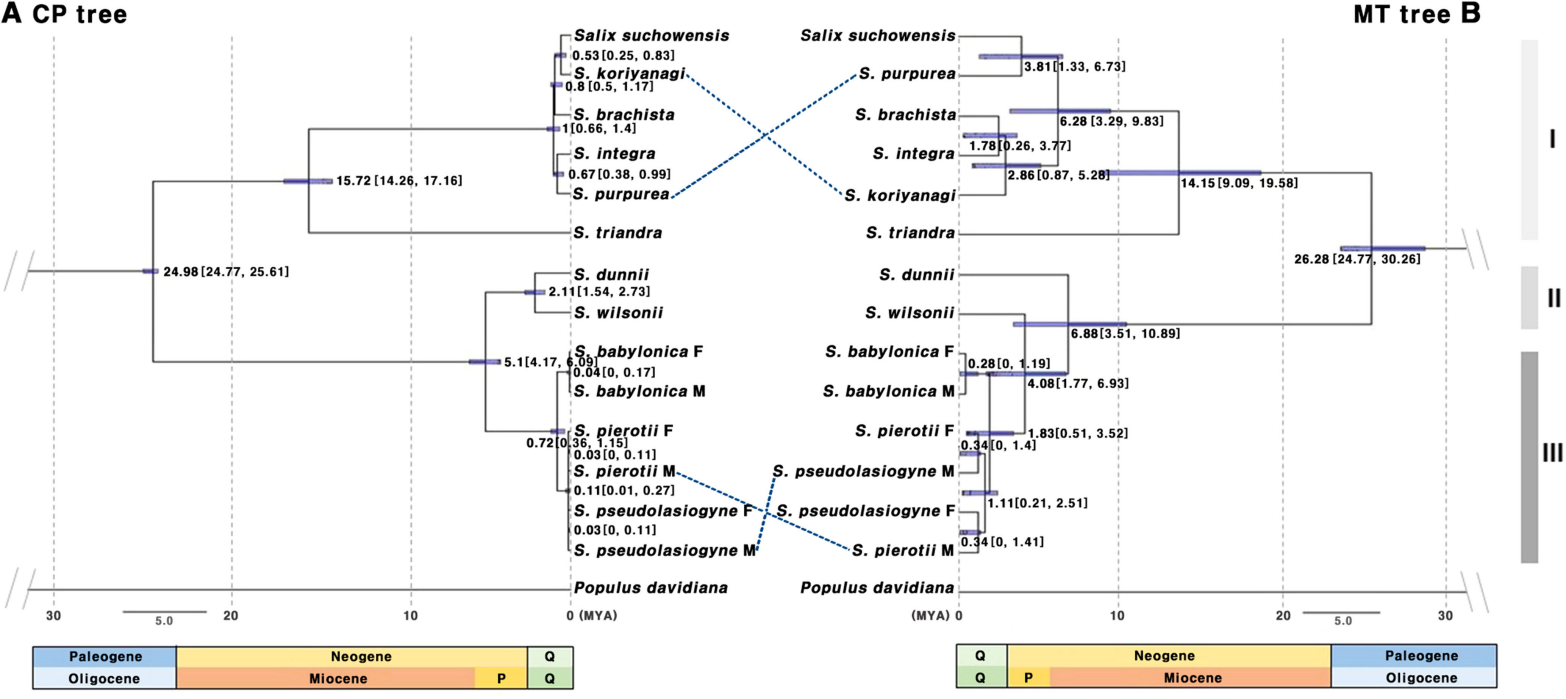
Chronogram based on protein-coding gene sequences from 11 *Salix* species. **(A)** Tree constructed using chloroplast genes. **(B)** Tree constructed using mitochondrial genes. The molecular clock trees were generated using the BEAST program. Numbers on the branches represent the mean divergence times, and the blue bars indicate the 95% highest posterior density (HPD) intervals. Age estimates are presented in millions of years ago (MYA). Q, Quaternary includes the Holocene and Pleistocene; P, Pliocene; Group I (G1): other sections, Group II (G2): sect. *Wilsonia*, Group III (G3): sect. *Salix*; M, Male; F, Female.

Divergence time estimation indicated that the first divergence among the 11 *Salix* species occurred during the Oligocene, approximately 24–26 MYA (**Figure 3**). Consistent topologies were observed for ML and BI trees, indicating a similar divergence time among the *Salix* species. Based on the tree-based analysis, species were grouped into three categories: Group I (G1), including species from the sections *Amygdalinae*, *Caesiae*, *Haoanae*, *Helix*, and *Lindleyanae*; Group II (G2), containing species from the section *Wilsonia*; and Group III (G3), consisting of species from the section *Salix*. The chloroplast-based tree revealed that Group G1 diverged approximately 15.72 MYA (Miocene, 95% highest posterior density or HPD: 14.26–17.16 MYA), with most divergences within G1, G2, and G3 occurring during the Quaternary period. In contrast, the mitochondrial-based tree revealed that G1 and G2 began diverging in the Miocene, with subsequent divergences occurring during the Pliocene and Quaternary. Both the chloroplast- and mitochondrial-based trees indicated that G1, G2, and G3 formed monophyletic clades, although some topological discrepancies were observed between the trees for certain species. Specifically, in G1, discrepancies between *S. koriyanagi* and *S. purpurea* were observed. Similarly, in G3, discrepancies were found between the male (M) individuals of *S. pierotii* (M) and those of *S. pseudolasiogyne* (M). These discrepancies may reflect differences in phylogenetic topology between the two organelle genomes, potentially attributable to recent divergence, hybridization, or distinct evolutionary dynamics. Moreover, the mitochondrial-based tree further supported that, within G3, *S. pierotii* and *S. pseudolasiogyne* were genetically closer to each other than to *S. babylonica*.

### 3.4 Selection pressure analysis

**Figure 4:**
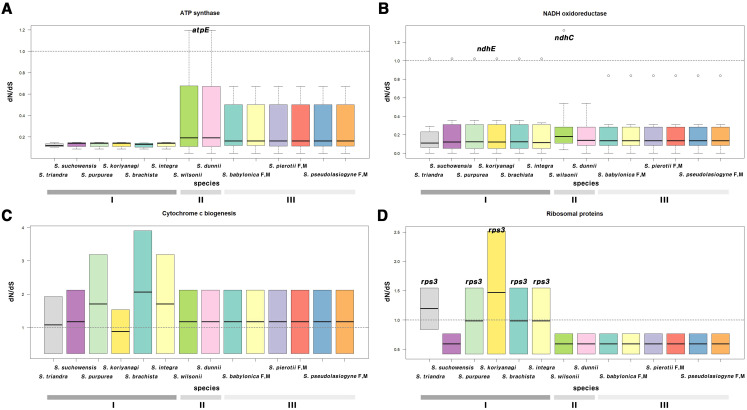
Boxplot of dN/dS ratios for chloroplast and mitochondrial genes, categorized by functional groups. Genes related to **(A)** ATP synthase, **(B)** NADH oxidoreductase, **(C)** cytochrome c biogenesis, and **(D)** ribosomal proteins. **(A, B)** represent chloroplast genes, while **(C, D)** represent mitochondrial genes. The horizontal line inside the box represents the median. The box extends from the first quartile (Q1) to the third quartile (Q3), and outliers are shown as individual points not connected by vertical lines above or below the box. Group I (G1): other sections, Group II (G2): sect. *Wilsonia*, Group III (G3): sect. *Salix*; M, Male; F, Female.

G1, G2, and G3, categorized by section, clustered similarly in terms of selection pressure (**Figure 4**; **Supplementary Figures 10**, **11**; **Supplementary Tables 12**, **13**). Most genes had ω < 1, indicating purifying selection; only a few genes exhibited ω > 1. In the chloroplast genome, genes such as *atpE*, *ndhC*, and *ndhE* showed elevated ω values, with *ndhE* in G1 (ω = 1.0223) and *atpE* in G2 (ω = 1.1952) showing signs of positive selection (**Figures 4A, B**). In the mitochondrial genome, genes that exhibited signals of positive selection included *ccmFc*, *cox3*, *rps3*, *matR*, and *nad4* (**Supplementary Table 13**). Notably, two genes, namely *matR* and *ccmFc*, exhibited positive selection across all 11 species (**Figure 4C**). In *S. triandra* of G1, *nad4* had an ω of 1.1731, whereas *cox3* and *rps3* showed ω > 1 in most G1 species (**Figure 4D**). Among mitochondrial genes, *ccmFc* showed relatively high ω values ranging from 1.9293 to 3.9009, and *rps3* ranged from 1.5445 to 2.5163, and *cox3* showed a value of 3.9641.

### 3.5 Gene transfer from chloroplast to mitochondria

**Figure 5:**
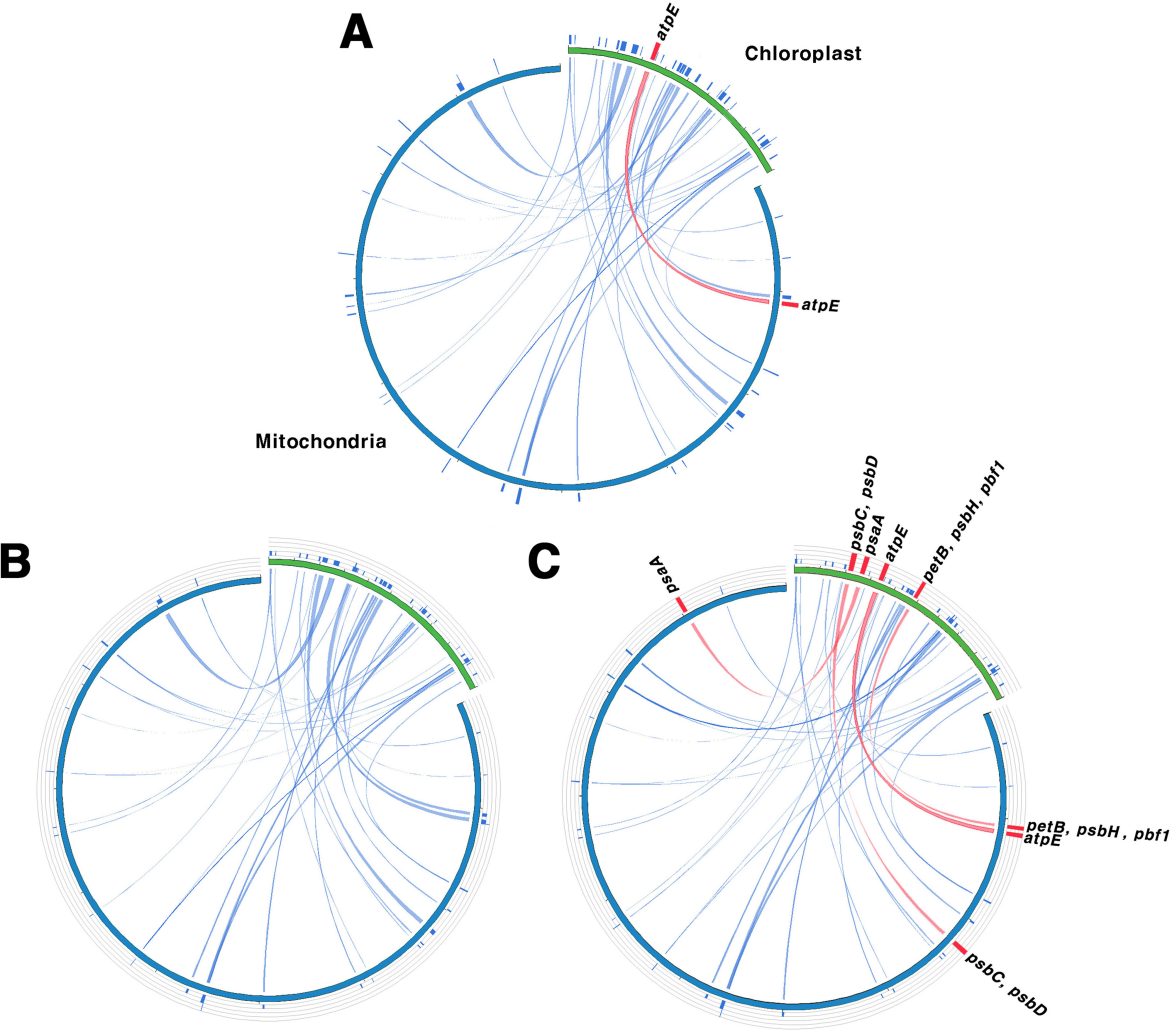
Homologous sequences shared between the chloroplast and mitochondrial genomes. **(A–C)** serve as representatives for Groups I, II, and III, respectively. Green indicates the chloroplast genome, while blue indicates the mitochondrial genome. Blue lines represent homologous fragments, and the red lines show complete transferred genes. Group I (G1): other sections, Group II (G2): sect. *Wilsonia*, Group III (G3): sect. *Salix*.

The sequence similarity between the chloroplast and mitochondrial genomes was consistent among the two tree-based analyses, grouping the species into G1, G2, and G3 (**Figure 5**). This grouping was indicative of complete gene transfer events. Chloroplast homologous fragments, ranging from 31 to 43 bp, were also present in the mitochondrial genome, indicating that numerous sequence fragments have undergone gene transfer (**Supplementary Tables 14**, **15**). We classified gene transfer into two categories: partial transfers, where only a portion of the gene sequence was transferred, and complete transfers, where the entire gene sequence was transferred. For protein-coding genes, there were about 12–14 partial transfers and between 0–7 complete transfers. In G1, only *atpE* underwent complete gene transfer, whereas G2 exhibited only partial transfers and no complete gene transfers. In contrast, G3 exhibited the highest number of complete gene transfers, with seven genes—*psaA*, *psbC*, *D*, *H*, *pbf1*, *petB*, and *atpE*—being fully transferred. Additionally, after removing duplicate sequences, we identified 10 to 11 cases of tRNA gene transfer across the three groups.

## 4 Discussion

### 4.1 Characteristics of organelle genomes in three *Salix* species

The chloroplast genome size observed for the three *Salix* species were consistent with previously reported ranges for the genus *Salix* (Wang and Yang, 2016; Wu and Li, 2022; Yang et al., 2022). The genes identified in the chloroplast genomes in the present study closely resembled those reported previously in *S. babylonica* (Wang and Yang, 2016), with the exception of one gene. Specifically, the *ycf15* was absent due to pseudogenization, a phenomenon commonly observed in many plant species due to early stop codons (Steane, 2005; Li et al., 2017). The conserved structure and composition of the chloroplast genome highlights its critical role in photosynthesis, plant growth, and stress responses (Wicke et al., 2011; Song et al., 2021).

The mitochondrial genomes of the three analyzed *Salix* species shared a consistent circular structure, which aligns with previously reported mitochondrial genomes in *Salix* species (Han et al., 2022; Ye et al., 2017). These genomes comprised 59 genes, which was within the range (55–59 genes) reported previously for other *Salix* species (Han et al., 2022; Ye et al., 2017). This relatively small gene set is a characteristic feature of plant mitochondrial genomes, which have undergone significant gene loss or gene transfer to the nuclear genome over the course of evolution (Adams et al., 2002). Despite the reduced gene content, the remaining genes are crucial for vital cellular functions, including energy metabolism. The genome sizes of the 11 *Salix* mitochondrial genomes were different, ranging from 598,970 bp in *S. purpurea* to 735,196 bp in *S. triandra*. These differences in genome length were likely due to structural variability, a common feature of plant mitochondrial genomes. This variability indicates their ability to undergo structural rearrangements, including duplications and deletions, and highlight the ability of plant mitochondria to adapt to diverse environmental conditions, thereby reinforcing the critical role played by plant mitochondrial genomes (Mackenzie and McIntosh, 1999; Morley and Nielsen, 2017).

### 4.2 Repeat element contributions to genome length

We identified 90–109 SSRs in the chloroplast genomes and 514–619 SSRs in the mitochondrial genomes (**Figure 2**). The prevalence of A/T repeat sequences indicates a higher occurrence of poly(A) and poly(T) sequences, which is a common pattern observed in many plant species (Kuntal and Sharma, 2011; Martin et al., 2013; Curci et al., 2015). The number of SSRs identified in the chloroplast genomes of *Salix* species in this study falls within the range previously reported in a comparative analysis of 10 *Salix* species, which identified 80–111 SSRs with repeat lengths of 30–35 bp (Yuan et al., 2025). Similarly, our mitochondrial SSR results are consistent with earlier findings, where species such as *S. wilsonii*, *S. cardiophylla*, *S. paraflabellaris*, and *S. suchowensis* were reported to contain around 608 SSRs and large dispersed repeats exceeding 1,000 bp (Ye et al., 2017). These results support the observation that mitochondrial genomes harbor longer repeat sequences than chloroplast genomes. However, when comparing the proportion of repetitive sequences relative to the chloroplast or mitochondrial genome length, both organelles exhibited similar average values of approximately 3%. This suggests that the greater abundance of repeats in the mitochondrial genome is primarily attributable to its larger genome size. Frequent DNA exchanges with the nuclear and chloroplast genomes, the presence of large introns, and extensive non-coding regions are also likely to have contributed to the overall expansion of the mitochondrial genome (Timmis et al., 2004; Dong et al., 2018). Repeat sequences also play a crucial role in shaping the structure of organelle genome. The abundance of repeat sequences is closely associated with recombination, which plays a critical role in the evolutionary dynamics of organelle genomes. Furthermore, repeat units and their associated sequences often induce structural changes within organelle genomes (Sinn et al., 2018; Varré et al., 2019; Zou et al., 2022).

### 4.3 Understanding phylogeny and evolutionary dynamics in *Salix* using organelle genomes

Based on the phylogenetic tree-based analyses, the 11 *Salix* species were grouped into two main subgenera, *Salix* and *Vetrix*, which is consistent with previous studies (Wu et al., 2015; Ren et al., 2022). While *Salix* is generally classified into three major subgenera—*Salix*, *Chamaetia*, and *Vetrix* (Skvortsov, 1999)—we found that *Chamaetia* appeared to be included within *Vetrix*. This supports the findings of recent studies, which suggest that *Chamaetia* and *Vetrix* form a single clade (Wu et al., 2015; Wagner et al., 2018, 2020, 2021). Furthermore, a recent molecular phylogenomic study proposed an updated classification that recognizes five subgenera within *Salix*, namely *Salix*, *Urbaniana*, *Triandrae*, *Longifoliae*, and *Vetrix* (Chen et al., 2025). The chloroplast-based trees showed consistent topologies across both ML and BI methods, whereas the mitochondrial-based trees exhibited some topological discrepancies (**Supplementary Figures 8**, **9**). Such discrepancies between the ML and BI trees may arise from differences in the statistical models employed by each method. Several studies have discussed potential causes for these topological inconsistencies, including differences in genomic regions analyzed, methodological approaches, and model assumptions (Huelsenbeck, 1995; Sullivan and Joyce, 2005; Som, 2014). Among the species analyzed, *S. triandra* occupies a unique position at the boundary between the *Salix* and *Vetrix* subgenera, and its phylogenetic placement has long been debated (Ren et al., 2022; Wu and Li, 2022; Trybush et al., 2008). This debate arises from the similar heterostyly system shared with other *Vetrix* species, as well as the genetic overlap between the *Salix* and *Vetrix* subgenera, further complicated by potential hybridization events (Li et al., 2020; Gulyaev et al., 2022). Recent phylogenomic studies have suggested recognizing *Triandrae* as a distinct subgenus (Chen et al., 2025). In our study, the three male and female individuals analyzed formed a monophyletic group, with *S. pierotii* and *S. pseudolasiogyne* clustering closely together. The organelle genome sequences of these two species exhibited minimal variation, with the chloroplast genome showing insufficient divergence to resolve species-level relationships. This limited divergence is not unique to these species but rather reflects the overall high conservation of chloroplast genomes in the *Salix* species, which can likely be attributed to strict functional constraints, low mutation rates, and limited recombination. This pattern is consistent with reports that chloroplast genome variation in *Salix* is much lower compared to other angiosperms (Wagner et al., 2021b), further emphasizing the extremely conserved nature of the chloroplast genome within the genus. This strong conservation reduces phylogenetic resolution, posing challenges for species delimitation and evolutionary inference. Given these limitations, incorporating nuclear or mitochondrial genome data may provide additional resolution and improve species-level phylogenetic studies within this genus.

In light of the close phylogenetic relationships among the three *Salix* species—*S. pierotii*, *S. babylonica*, and *S. pseudolasiogyne*—we hypothesized that these species may have experienced historical gene flow or genetic admixture due to their genetic relatedness. Hence, we estimated the divergence times of 11 *Salix* species and analyzed their evolutionary relationships, focusing on the chloroplast and mitochondrial genomes (**Figure 3**). The first divergence among the 11 *Salix* species was estimated at 24.98 MYA (95% HPD: 24.77–25.61) for the chloroplast genome and 26.28 MYA (95% HPD: 24.77–30.26) for the mitochondrial genome, suggesting that the two organelles diverged within a very similar time frame. Beyond the initial divergence, the internal branching patterns of the chloroplast and mitochondrial phylogenies differed. The mitochondrial tree displayed longer internal branches among species, indicating greater sequence divergence and lineage differentiation. In contrast, the chloroplast tree exhibited shorter branches and tighter species clustering, suggesting either more recent divergence or stronger sequence conservation (**Figure 3**). We also observed discrepancies between G1 and G3, likely owing to differences in evolutionary pressures and evolutionary rates affecting each genome. Chloroplast genomes are typically more conserved and exhibit lower genetic diversity compared to mitochondrial and nuclear genomes. This is primarily due to their maternal inheritance and their essential role in photosynthesis, which contribute to maintaining a stable genomic structure over time (Zurawski and Clegg, 1987; Wicke et al., 2011). The slower evolution observed in *Salix* can be explained by its characteristics as a woody plant, including longer life cycles and slower growth rates. In contrast, plant mitochondrial genomes, although characterized by low mutation rates, exhibit greater structural variability compared to chloroplast genomes. This encompasses gene rearrangements, structural variations, and frequent recombination events. Such structural flexibility allows plant mitochondrial genomes to adapt to various environmental pressures, playing a key role in shaping the evolutionary patterns of plant species. As a result, mitochondrial genomes contribute to broader processes of plant genome evolution and diversification (Palmer and Herbon, 1988; Gualberto and Newton, 2017; Sun et al., 2022), leading to evolutionary patterns distinct from those of the more stable chloroplast genomes.

Within the mitochondrial-based phylogeny of G3, our data indicate potential gene flow or genetic admixture between *S. pierotii* and *S. pseudolasiogyne*, estimated at 4.08–6.88 MYA, before the divergence of *S. wilsonii*. The observed clustering of these species in the mitochondrial tree could reflect recent divergence, historical or partial gene exchange, or unresolved polytomies resulting from rapid, nearly simultaneous divergences. Mitochondrial genomes, due to their dynamic structural evolution and tendency for gene exchange, are particularly responsive to external genetic influx, which can obscure species boundaries and complicate interpretations of evolutionary history (Zakharov et al., 2009; Yuan et al., 2023). In contrast, chloroplast genomes are highly conserved due to their essential role in photosynthesis, whereas mitochondrial genomes exhibit structural flexibility and frequent recombination (Gualberto et al., 2014). Despite their generally maternal inheritance, organelle genomes provide valuable, albeit sometimes partial, insights into evolutionary relationships. The observed patterns of gene flow or genetic admixture could be indicative of putative historical hybridization events, although organelle data alone cannot conclusively confirm hybridization. Nuclear genomic analyses, which capture biparental inheritance, are therefore crucial for validating these patterns and assessing the contribution of hybridization to species genomic composition (Greiner et al., 2015). Studies using nuclear markers suggest that hybridization is common in *Salix* and may help explain patterns of evolutionary change, potentially contributing to species diversification (Wagner et al., 2020; Barcaccia et al., 2014; Vašut et al., 2024). In our analysis of male and female individuals from the three studied species, sex-specific patterns were not evident; nonetheless, considering dioecy could provide additional context when interpreting gene flow and evolutionary dynamics in *Salix* (Nybakken and Julkunen-Tiitto, 2013).

### 4.4 Selective pressures on chloroplast and mitochondrial genes

The dN/dS analysis results showed that most genes in Group I (G1, section *Amygdalinae*, *Caesiae*, *Haoanae*, *Helix*, and *Lindleyanae*), Group II (G2, section *Wilsonia*), and Group III (G3, sections *Salix*) underwent neutral evolution or purifying selection, though several genes showed evidence of positive selection (**Figure 4**; **Supplementary Figures 10**, **11**; **Supplementary Tables 12**, **13**). A distinct separation was evident between the groups (**Figure 4A**), potentially shaped by environmental factors or habitat-specific characteristics. G1, which primarily comprised shrubs, may exhibit differences in photosynthetic efficiency, growth rate, or adaptation strategies compared to species in G2 and G3 (Santos et al., 2025). G2 comprised species from the subtropical regions of China, which appear to share similar environmental stresses or climatic conditions (Fang et al., 1999; Zurawski and Clegg, 1987; He et al., 2021b). Due to their geographic proximity and similar environmental conditions, G3 species appear to experience comparable selective pressures. As ATP synthase plays a critical role in plant photosynthesis (Westhoff et al., 1985), these ecological contexts may have contributed to the divergence in photosynthetic gene evolution. While growth form may influence certain aspects of adaptation, the consistent patterns observed across diverse *Salix* habitats highlight the predominant role of environmental conditions in shaping genomic evolution.

Chloroplast and mitochondrial genes showing evidence of positive selection were further examined to assess their functional significance and potential adaptive roles. Due to the demand for rapid activation, the *atpE* gene rate (Rott et al., 2011; Chotewutmontri and Barkan, 2016) exhibited a high evolutionary. The *ndh* gene family (from *ndhA* to *ndhK*) (Martin et al., 1996; Endo et al., 1999; Yamori et al., 2011; Li Q. et al., 2024), involved in the chloroplast electron transport chain, is crucial for adaptation to environmental stresses such as high light intensity and low temperatures. In G2, *atpE* appears to have undergone positive selection likely after its divergency from G3, ~5.1 MYA (95% HPD: 4.17–6.09). Similarly, the *ndhC* in *S. wilsonii* underwent positive selection ~2.11 MYA (95% HPD: 1.54–2.73) after diverging from *S. dunnii*. Positive selection for *ndhE* in G1 was estimated to have occurred between 15.72 and 24.98 MYA. Most mitochondrial genes had ω values <1, though some genes exhibited different patterns. The *matR* gene (Adams et al., 2002), essential for mitochondrial function, showed evidence of positive selection across all species in G1, G2, and G3. Additionally, *ccmFc* (Giegé et al., 2004), which is essential for cytochrome c maturation, consistently exhibited ω >1 (mean = 2.5) across all species. No additional positively selected mitochondrial genes were identified in G2 or G3. In G1, *nad4* gene in *S. triandra* is a subunit of NADH dehydrogenase and is thought to have adapted to environmental conditions post divergence ~14.15 MYA (95% HPD: 9.09–19.58). Additionally, the *rps3* (Passmore et al., 2007) in mitochondrial ribosomes, essential for protein translation initiation, exhibited a dN/dS ratio >1.5 in all G1 species except *S. suchowensis*, indicating positive selection. Interestingly, *S. suchowensis* shared some gene patterns with species in G2 and G3, suggesting exposure to similar environmental pressures. These results indicate that both chloroplast and mitochondrial genes have undergone group-specific adaptation to environmental pressures, highlighting the diverse evolutionary dynamics of the *Salix* species.

Some mitochondrial genes, particularly *ccmFc, cox3* and *rps3*, exhibited relatively higher dN/dS ratios than chloroplast genes, suggesting stronger positive selection. Hence, mitochondrial genomes may experience stronger selective pressures than chloroplast genomes, likely due to their distinct functions and unique genetic and metabolic characteristics (Jiang et al., 2018). Mitochondrial genes often show high variability in non-synonymous substitution rates among genes and lineages (Lynch et al., 2006; Richardson et al., 2013; Smith and Keeling, 2015). In contrast, chloroplast genomes accumulate relatively few non-synonymous substitutions, reflecting strong purifying selection to maintain essential photosynthetic functions. This difference may explain the stronger signals of positive selection observed in mitochondrial genes in this study. The contrasting selective pressures between the two organelles suggest that mitochondrial genes are subject to more dynamic evolutionary forces, likely driven by environmental adaptation and metabolic demands. Moreover, the evidence of positive selection in both organelles indicates the crucial role of cytoplasmic genome evolution in shaping the ecological adaptability of *Salix*, potentially contributing to its success across diverse environmental conditions.

### 4.5 Evolutionary patterns of chloroplast gene migration to mitochondria

Gene transfer between organelle and nuclear genomes as well as between different organelle genomes is a frequent and well-documented phenomenon in plants (Nguyen et al., 2000; Bergthorsson et al., 2003; Timmis et al., 2004). These transfers are crucial in genome evolution, contributing to genetic diversity and complexity (Huang et al., 2005; Yue et al., 2022). To investigate gene transfer relationships between chloroplast and mitochondrial genomes in *Salix*, we analyzed homologous fragments (**Figure 5**; **Supplementary Tables 14**, **15**). Our analysis grouped the 11 *Salix* species into three distinct groups. G1 corresponded to subgenus *Salix* s.str., G2 to subgenus *Protitea*, and G3 to species primarily classified under subgenus *Chamaetia*/*Vetrix* (Wu et al., 2015). Despite its ambiguous taxonomic status, *S. triandra*, was assigned to G3 due to its clustering with *Chamaetia*/*Vetrix* species in earlier phylogenetic analyses (Yuan et al., 2025). All three groups exhibited homologous sequences and evidence of chloroplast-derived sequences in the mitochondrial genome, indicating potential gene transfer events. These events likely predated the divergence of G1, G2, and G3, which is estimated to have occurred around 24.95–26.28 MYA. Among the transferred sequences, tRNA genes were the most frequently transferred. Many of these transferred sequences, including tRNA genes, appear to have undergone pseudogenization (Straub et al., 2013; Wee et al., 2022). Although the transfer events likely occurred before the divergence of these groups, the number of retained transferred genes varied among them: G3 contained seven transferred genes, whereas G1 and G2 had only 0–1. This suggests that G3 may have preserved or accumulated more transferred sequences, possibly due to distinct evolutionary pressures, greater genomic plasticity, or adaptation to specific environmental conditions.

These three groups indicated how environmental factors, gene transfer, and evolutionary processes have shaped genetic diversity in *Salix*. It also facilitates better understanding of how gene flow interacts with ecological pressures. Our findings indicate that *Salix* species have adapted to common environmental conditions—such as climate, habitat, and interspecific competition—resulting in divergent yet interconnected lineages. Closely related species, including *S. pierotii*, *S. babylonica*, and *S. pseudolasiogyne*, share overlapping habitats and similar reproductive strategies, which likely facilitated gene flow or genetic admixture. Additionally, recent divergence, continued hybridization, and introgression likely blur species boundaries and promote genetic fluidity. Notably, gene flow was observed exclusively in the mitochondrial genome, suggesting distinct evolutionary constraints compared to the chloroplast genome.

## 5 Conclusion

In this study, we revealed contrasting evolutionary dynamics between chloroplast and mitochondrial genomes across 11 *Salix* species, thereby providing key insights into organelle genome evolution and intergenomic interactions. Our analysis identified distinct evolutionary patterns, with the mitochondrial genome showing relatively clearer phylogenetic separation among species than the chloroplast genome. This difference can be attributed to their functional roles: the chloroplast genome remained highly conserved due to its central role in photosynthesis, whereas the mitochondrial genome showed greater structural and evolutionary flexibility in response to environmental pressures. Moreover, several mitochondrial genes were found to be under stronger positive selection than their chloroplast counterparts, further illustrating their divergent evolutionary trajectories. We also detected evidence of gene transfer between chloroplast and mitochondrial genomes, highlighting functional interconnections between these organelles. These intergenomic exchanges may contribute to structural variations in the mitochondrial genome and influence the evolutionary dynamics of the *Salix* species. Notably, mitochondrial genome data suggested signals of potential gene flow or genetic admixture. These findings underscore the complementary role of mitochondrial genome analysis in resolving phylogenetic relationships and highlighting possible historical inter-species genetic exchange, especially given the limitations of the highly conserved chloroplast genome. Our integrative framework highlights the value of incorporating mitochondrial data into organelle genome studies and may serve as a foundation for broader applications in plant evolutionary biology and comparative genomics.

## Data Availability

The raw read data generated in this study are available in the Sequence Read Archive (SRA) under the BioProject accession number PRJNA1205789. Additionally, the newly sequenced chloroplast and mitochondrial genomes can be accessed in the NCBI database under the accession numbers PQ842549-54 and PQ873106-11.
