# Supplementary material for: Interactions between chloroplast and mitochondrial genomes in 11 Salix species
Source: Front Plant Sci. 2025 Nov 21;16:1693183. doi: 10.3389/fpls.2025.1693183 (PMC12678270; doi:10.3389/fpls.2025.1693183)
Supplement: Supplementary file 1 [file SupplementaryFile1.docx]

Supplementary Material

Interactions Between Chloroplast and Mitochondrial Genomes in *Salix* Species

Yeseul Kim^1^, Sumin Jeong^1^, Shukherdorj Baasanmunkh^1^, Youngmoon Kim^1^, Hyeok Jae Choi^1*^, Inkyu Park^1*^

^1^ Department of Biology, Changwon National University, Changwon 51140, Republic of Korea

*** Correspondence:**Inkyu Park and Hyeok Jae Choi
Email address: pik6885@cwnu.ac.kr, hjchoi1975@ cwnu.ac.kr
*These authors contributed equally to this work


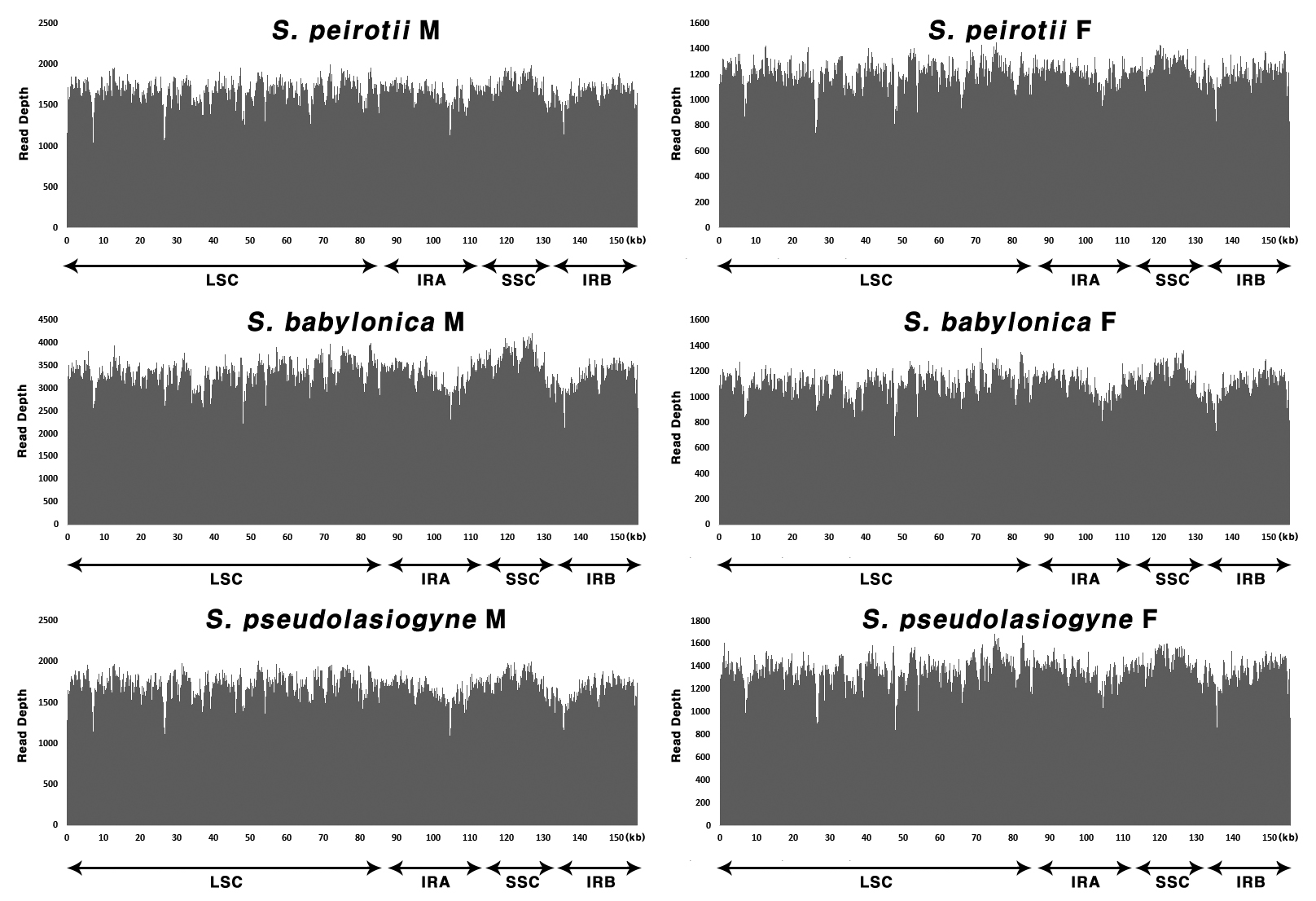


**Supplementary Figure 1.** Self-mapping and coverage of chloroplast genomes in *Salix pierotii*, *S.* *babylonica* and *S. pseudolasiogyne*. LSC: Large single copy, SSC: Small single copy, IR: Inverted repeat regions; M: Male, F: Female.


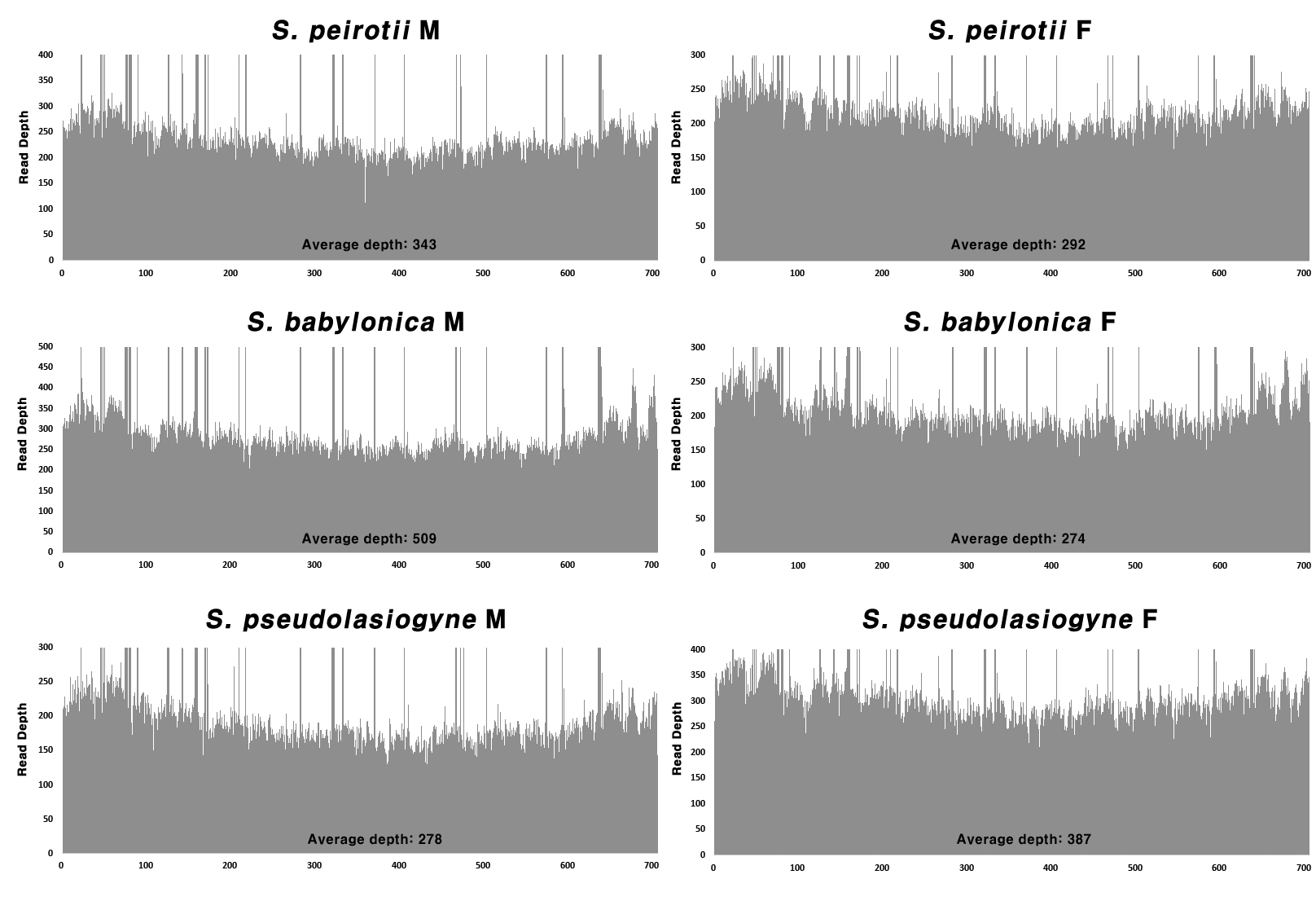


**Supplementary Figure 2.** Self-mapping and coverage of mitochondrial genomes in *Salix pierotii*, *S.* *babylonica* and *S. pseudolasiogyne*. M: Male, F: Female.


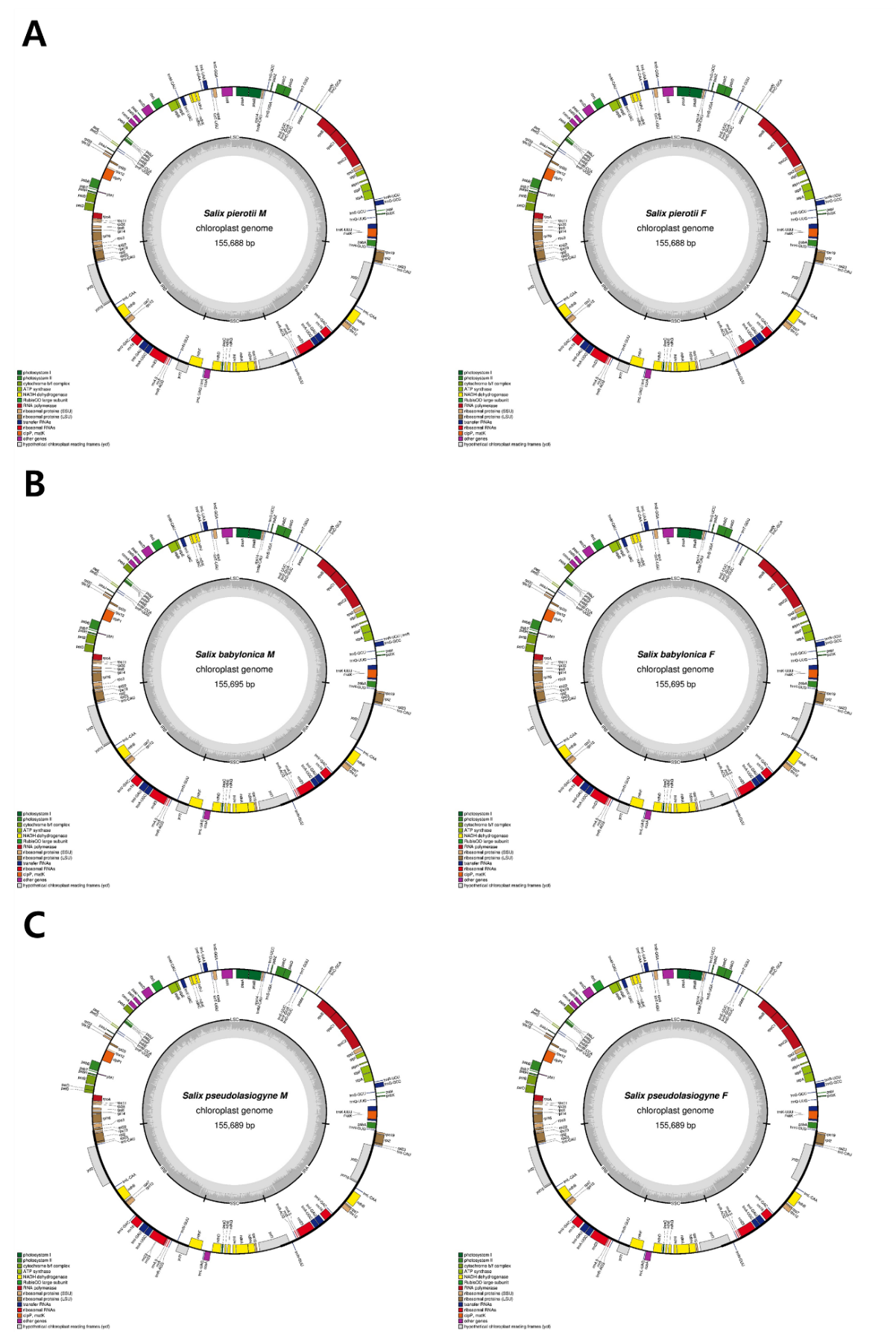


**Supplementary Figure 3.** Circular maps of the chloroplast genomes in male and female individuals of **(A)** *Salix pierotii*, **(B)** *S. babylonica*, and **(C)** *S. pseudolasiogyne*. M: Male, F: Female.

**
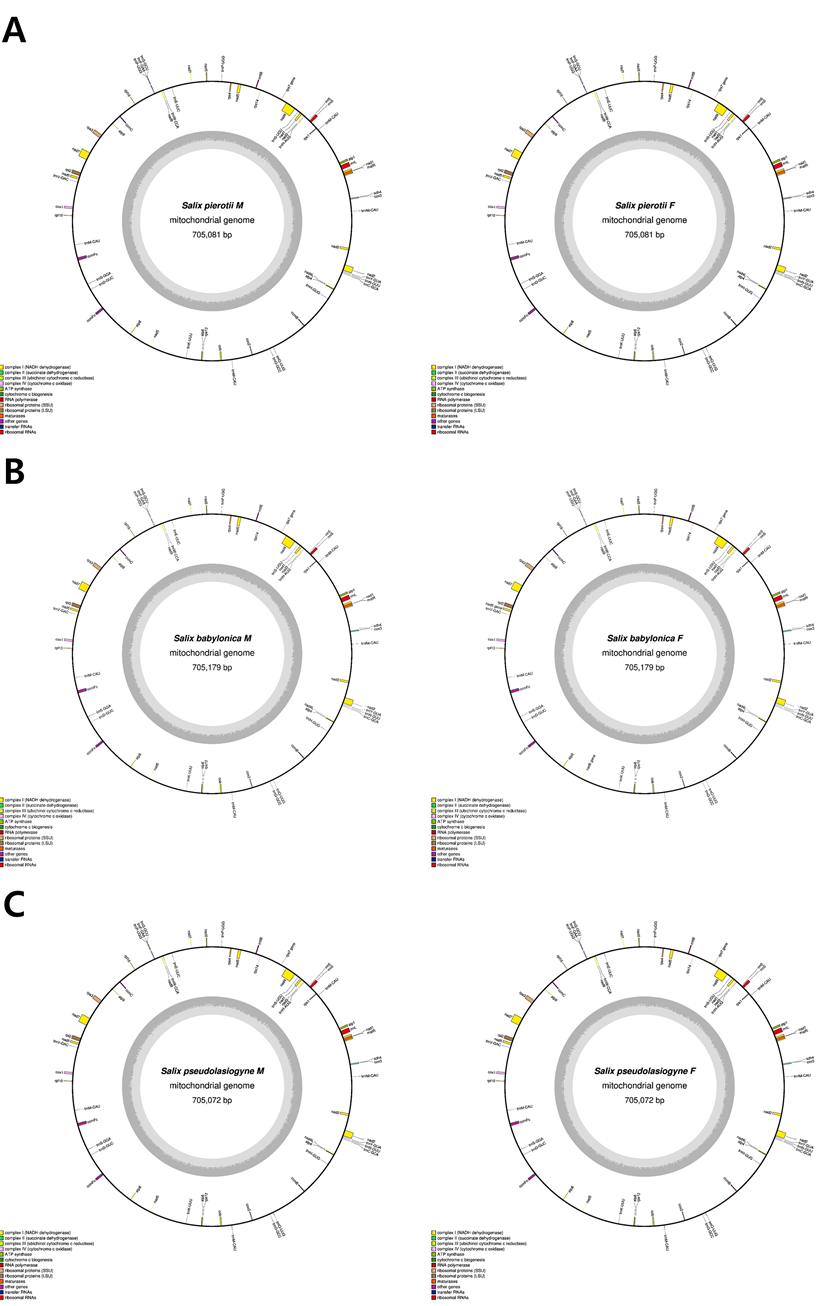
**

**Supplementary Figure 4.** Circular maps of the mitochondrial genomes in male and female individuals of **(A)** *Salix pierotii*, **(B)** *S. babylonica*, and **(C)** *S. pseudolasiogyne*. M: Male, F: Female.


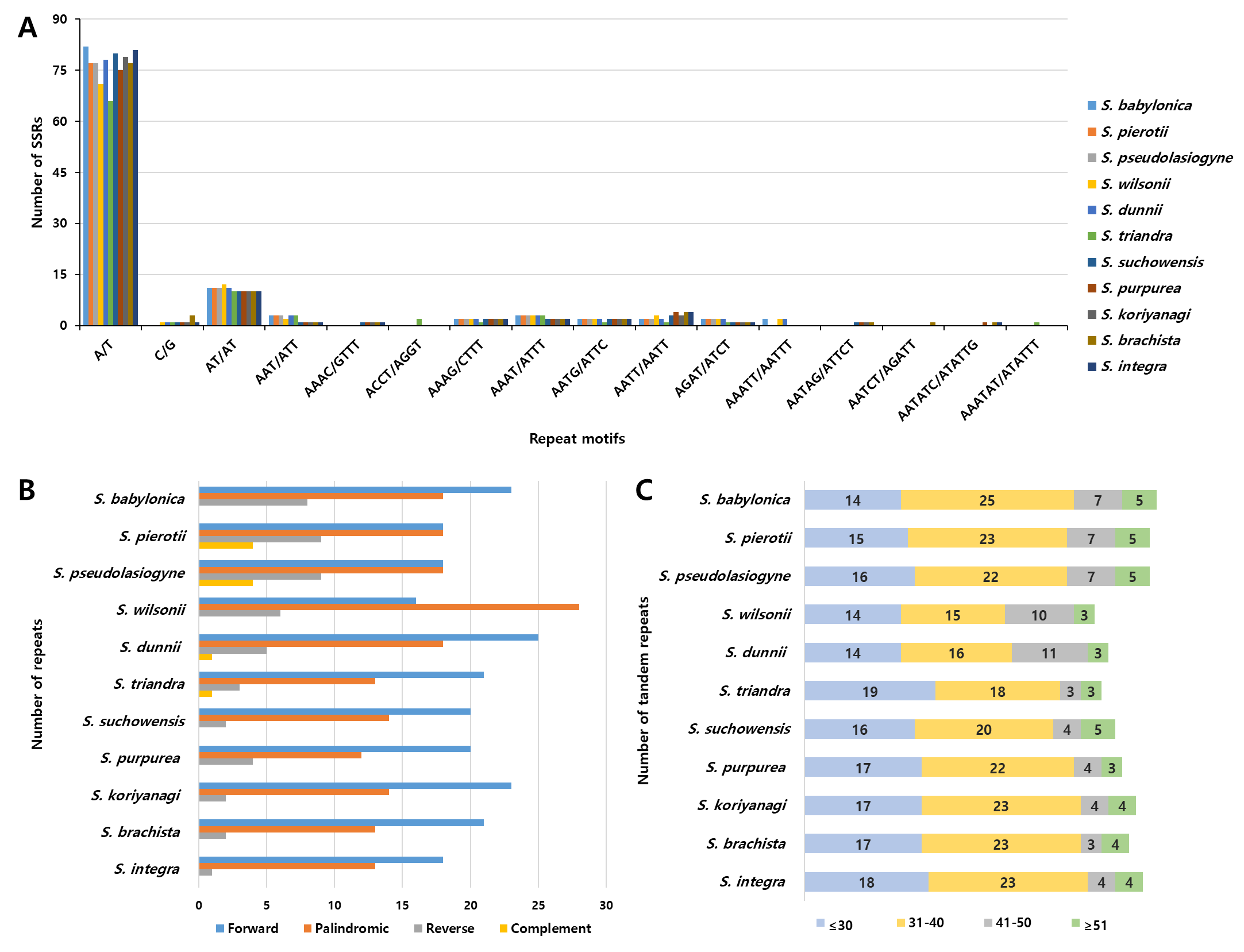


**Supplementary Figure 5.** Repeat sequences in the chloroplast genomes of 11 *Salix* species. **(A)** Identified SSR motifs. **(B)** Four types of repeat sequences. **(C)** Length frequency distribution of tandem repeats.


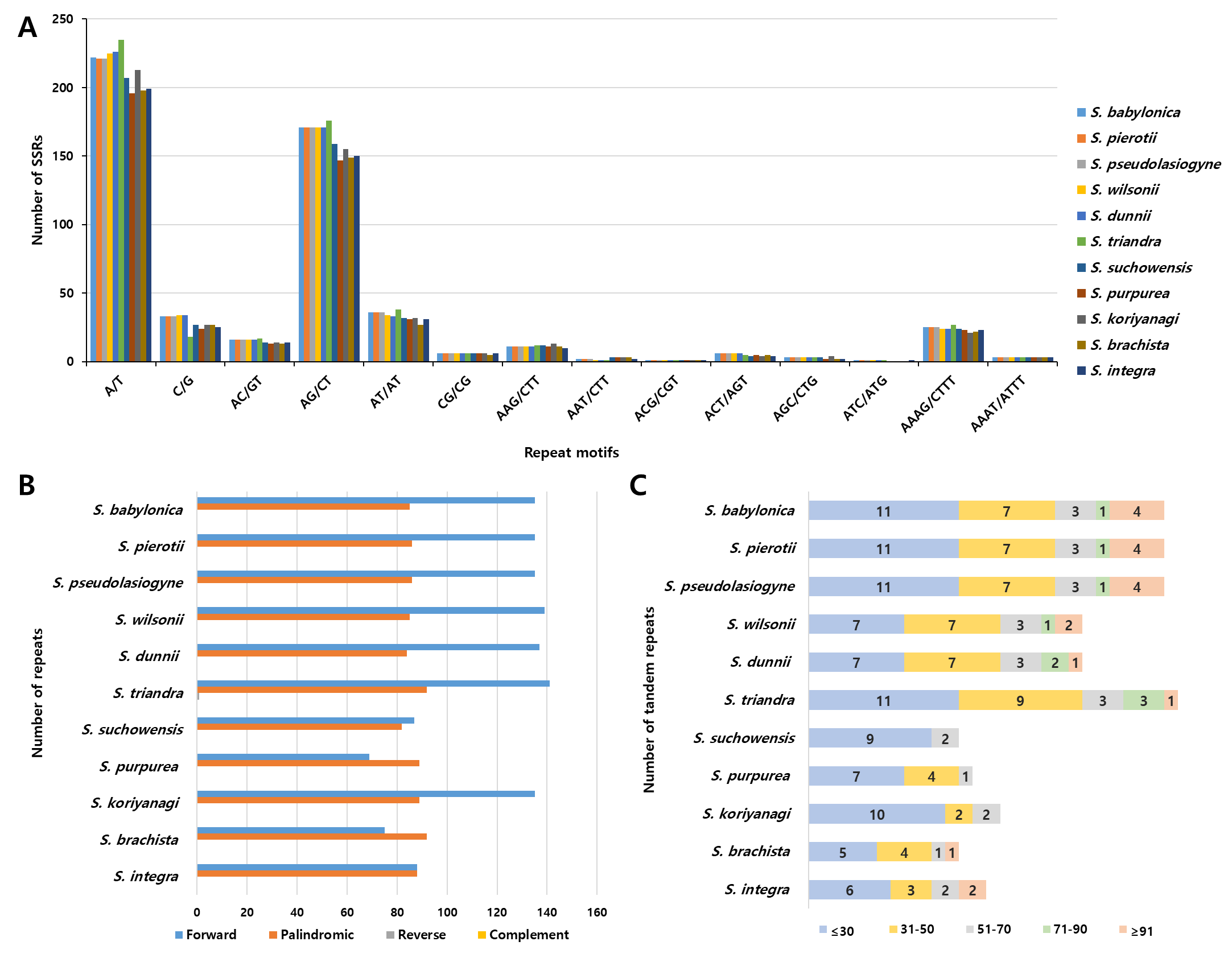


**Supplementary Figure 6.** Repeat sequences in the mitochondrial genomes of 11 *Salix* species. **(A)** Identified SSR motifs. **(B)** Four types of repeat sequences. **(C)** Length frequency distribution of tandem repeats.


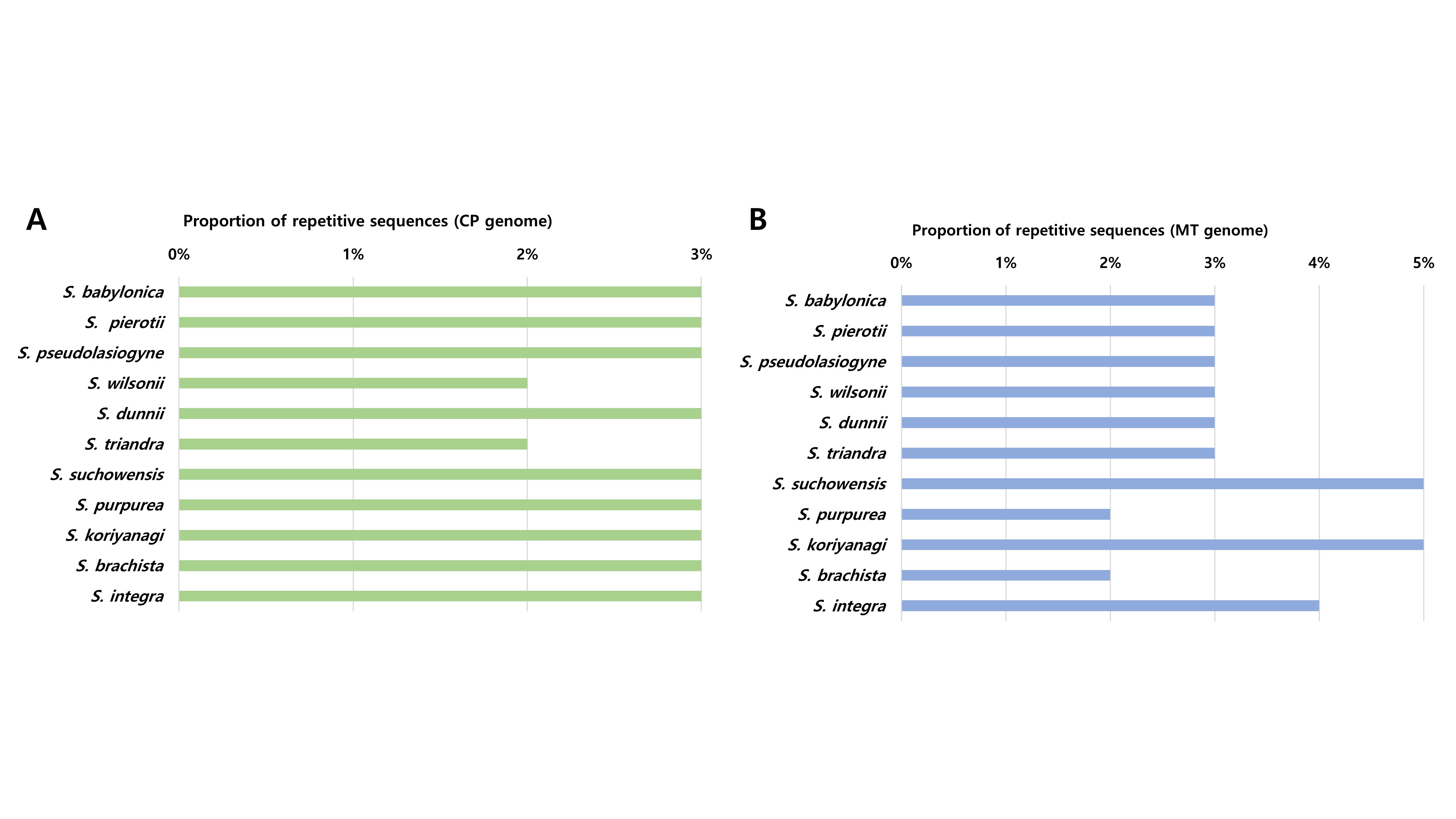


**Supplementary Figure 7.** Proportion of repetitive DNA content in *Salix* species. **(A)** Chloroplast genomes.
**(B)** Mitochondrial genomes. Each bar represents the percentage of repetitive sequences relative to the total chloroplast or mitochondrial genome length in each species.


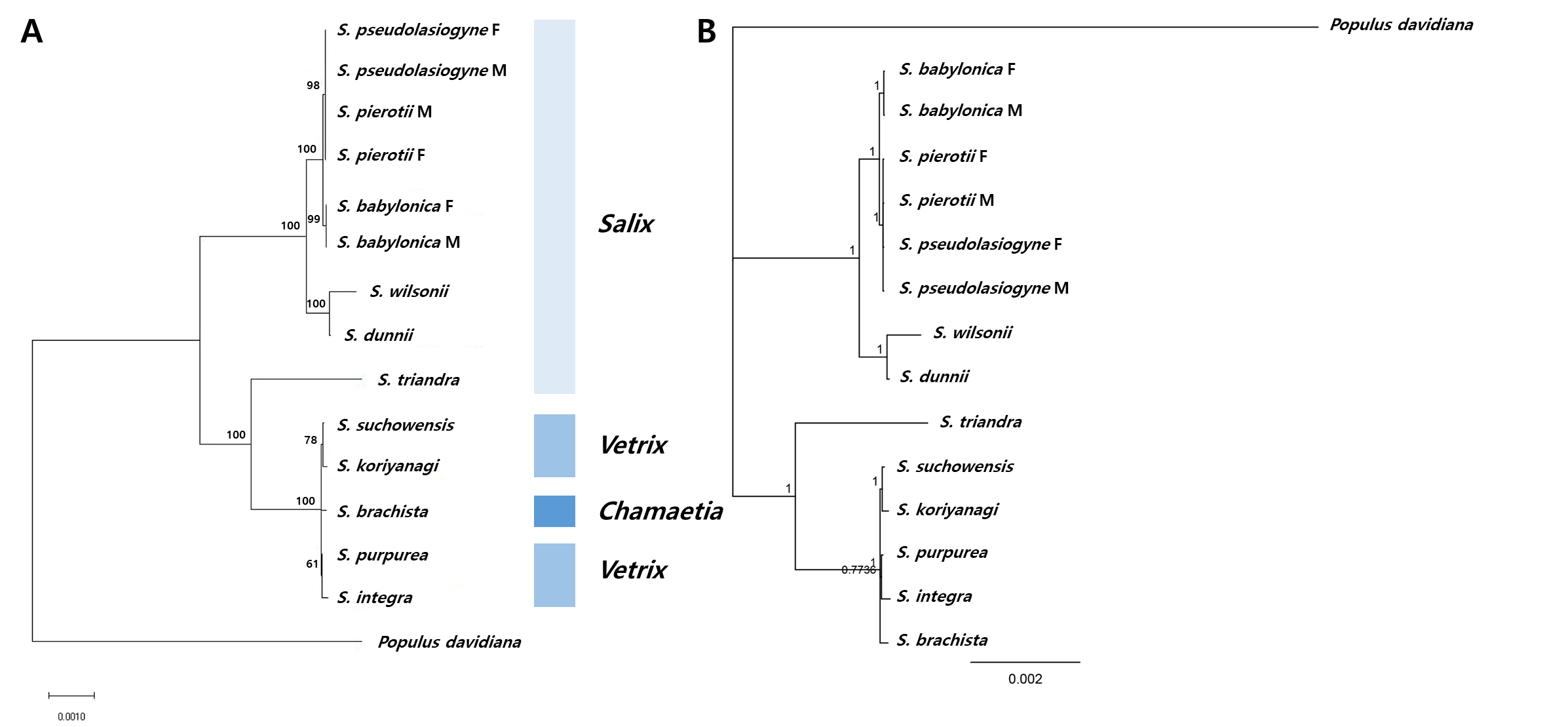


**Supplementary Figure 8.** Phylogenetic relationships of 11 *Salix* species based on chloroplast genes. **(A)** Phylogenetic tree constructed using the Maximum-Likelihood (ML) method, with numbers at each node representing bootstrap support values. **(B)** Phylogenetic tree constructed using the Bayesian Inference (BI) method, where numbers at each node indicate posterior probabilities (PP). M: male, F: female.


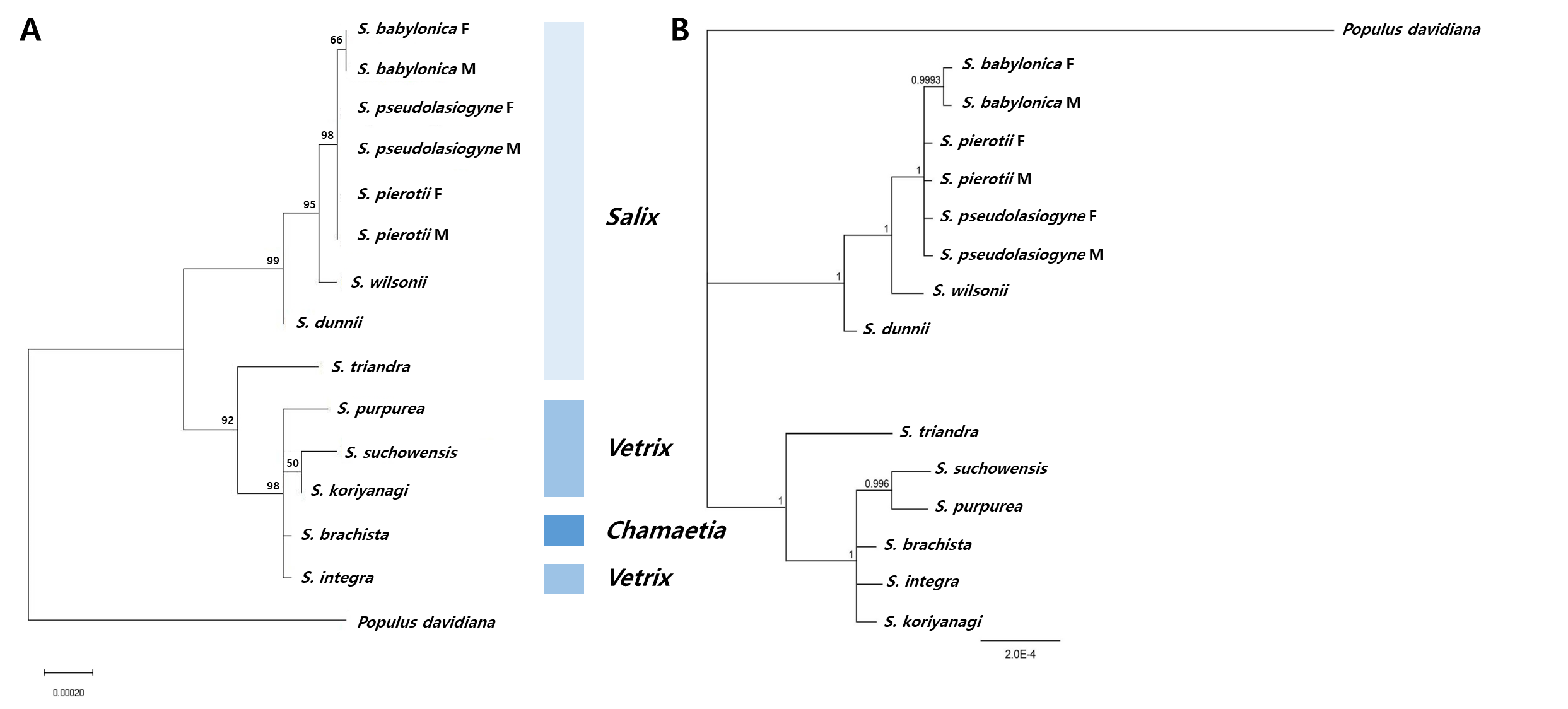


**Supplementary Figure 9.** Phylogenetic relationships of 11 *Salix* species based on mitochondrial genes. **(A)** Phylogenetic tree constructed using the Maximum-Likelihood (ML) method, with numbers at each node representing bootstrap support values. **(B)** Phylogenetic tree constructed using the Bayesian Inference (BI) method, where numbers at each node indicate posterior probabilities (PP). M: male, F: female.


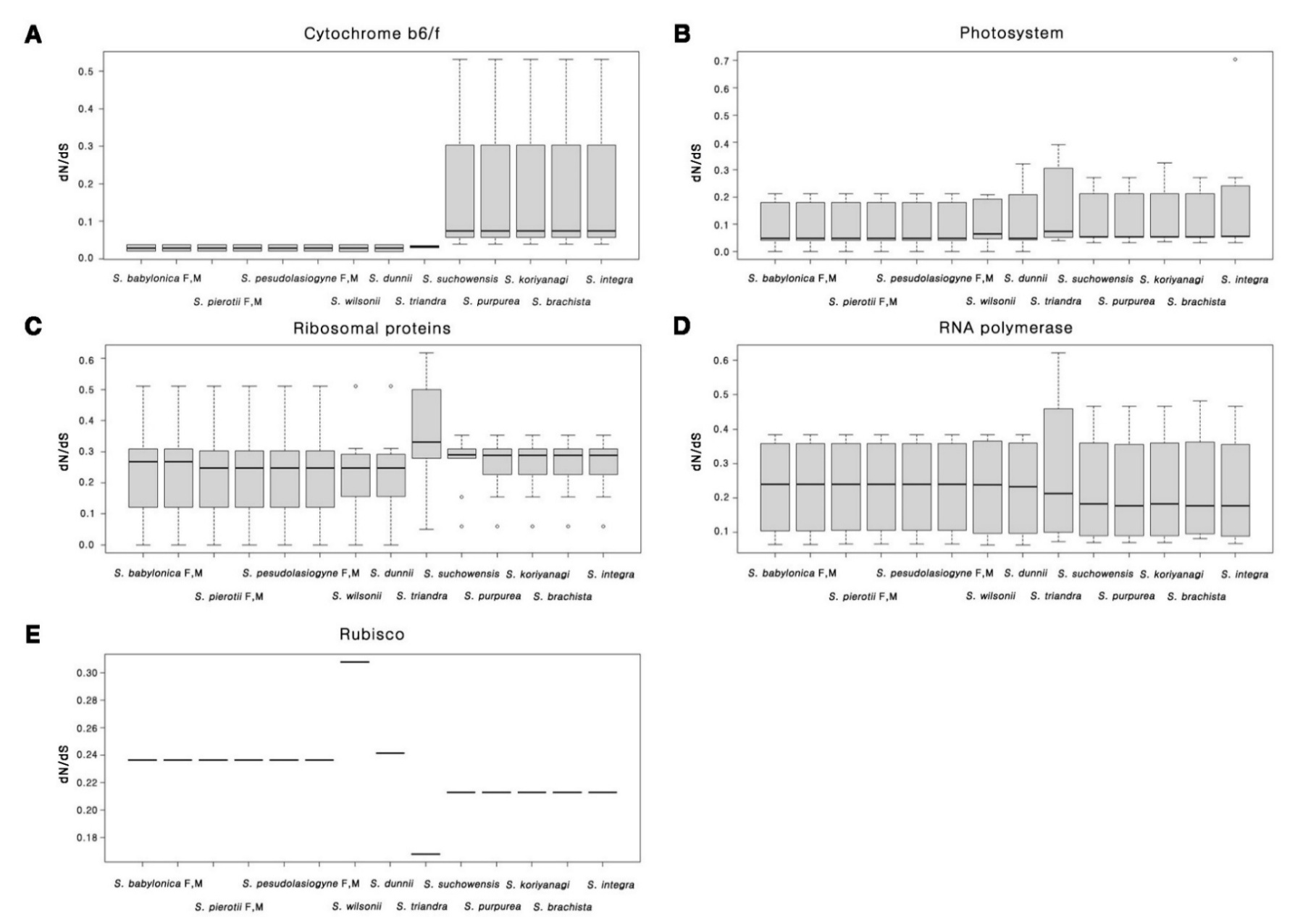


**Supplementary Figure 10.** Selection pressure analysis boxplot for chloroplast genes. Genes associated with **(A)** Cytochrome b6/f, **(B)** Photosystem, **(C)** Ribosomal proteins, **(D)** RNA polymerase, and **(E)** Rubisco. M: male, F: female. Detailed data are provided in Supplementary Table 12.


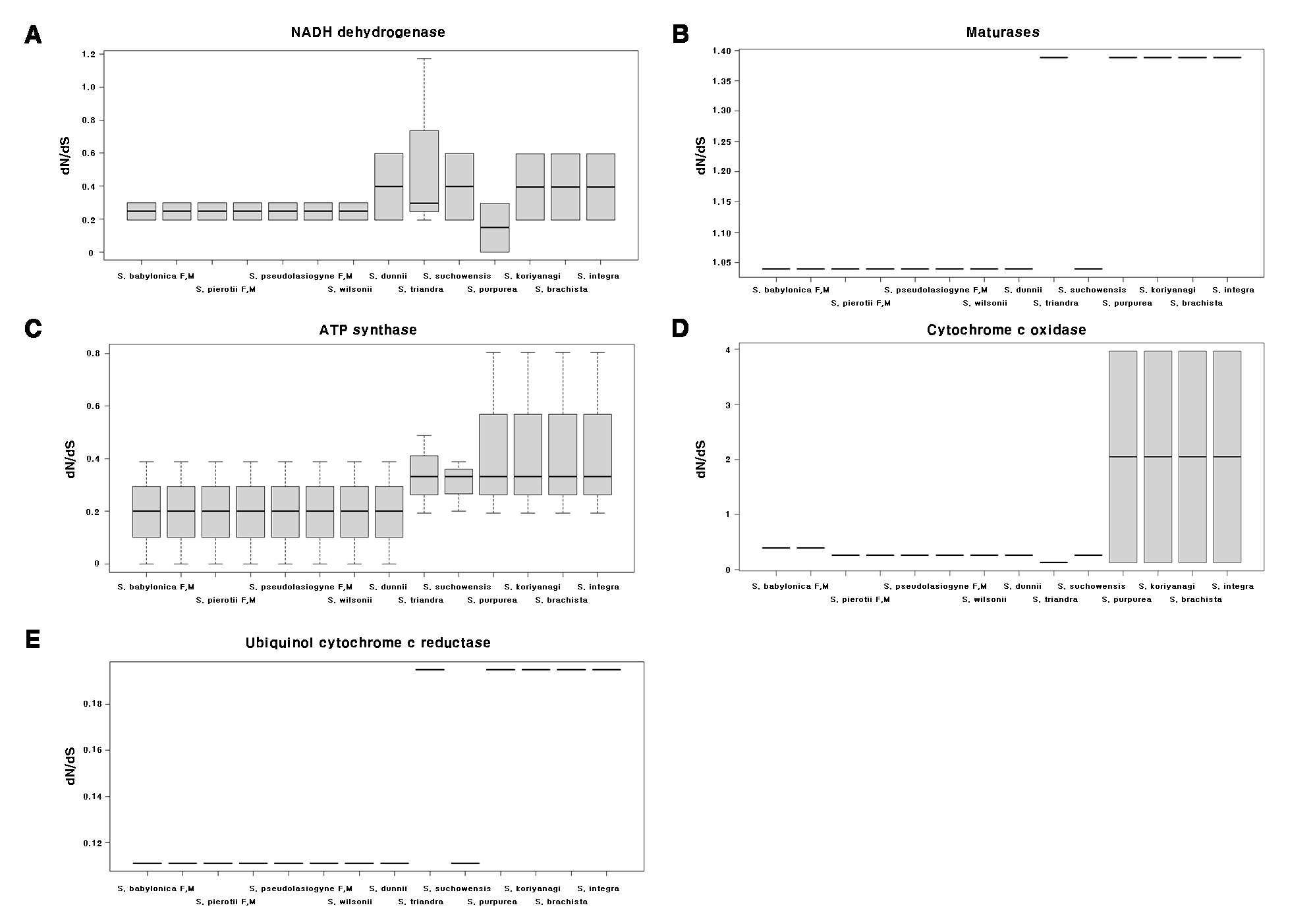


**Supplementary Figure 11.** Selection pressure analysis boxplot for mitochondrial genes. Genes associated with **(A)** NADH dehydrogenase, **(B)** Maturases, **(C)** ATP synthase, **(D)** Cytochrome c oxidase, and **(E)** Ubiquinol cytochrome c reductase. M: male, F: female. Detailed data are provided in Supplementary Table 13.

**Supplementary Table 1.** Collection information of male and female individuals of three *Salix* species. M: male, F: female.

| No. | Species | Collection information | Coordinates | Voucher number | Herbarium information |
| --- | --- | --- | --- | --- | --- |
| 1 | *S. pierotii* M | Hwayang-ri, Uiryeong-gun, Gyeongsangnam-do, Korea | 35°16'04.4"N 128°16'34.7"E | NG23042701 | Changwon National University |
| 2 | *S. pierotii* F | Hwayang-ri, Uiryeong-gun, Gyeongsangnam-do, Korea | 35°16'04.4"N 128°16'34.7"E | NG23042702 | Changwon National University |
| 3 | *S. babylonica* M | Samnak-dong, Sasang-gu, Busan, Korea | 35°10'17.2"N 128°58'27.5"E | SN23050401 | Changwon National University |
| 4 | *S. babylonica* F | Samnak-dong, Sasang-gu, Busan, Korea | 35°10'17.2"N 128°58'27.5"E | SN23050402 | Changwon National University |
| 5 | *S. pseudolasiogyne* M | Sarim-dong, Changwon-si, Gyeongsngnam-do, Korea | 35°14'26.1"N 128°41'51.4"E | CC23050101 | Changwon National University |
| 6 | *S. pseudolasiogyne* F | Sarim-dong, Changwon-si, Gyeongsngnam-do, Korea | 35°14'26.1"N 128°41'51.4"E | CC23050102 | Changwon National University |

**Supplementary Table 2.** Platform used for genome sequencing. M: male, F: female.

| Species | Reads | Bases | Sequencing platform |
| --- | --- | --- | --- |
| *S. pierotii* M | 92,212,016 | 13,924,014,416 | NovaSeq 6000 |
| *S. pierotii* F | 88,679,142 | 13,390,550,442 | NovaSeq 6000 |
| *S. babylonica* M | 99,006,432 | 14,949,971,232 | NovaSeq 6000 |
| *S. babylonica* F | 89,123,834 | 13,457,698,934 | NovaSeq 6000 |
| *S. pseudolasiogyne* M | 91,882,824 | 13,874,306,424 | NovaSeq 6000 |
| *S. pseudolasiogyne* F | 90,063,566 | 13,599,598,466 | NovaSeq 6000 |

**Supplementary Table 3.** Raw and trimmed read data. M: male, F: female.

| Species | Input reads | Trimmed reads | | Total raw bases | Trimmed bases | |
| --- | --- | --- | --- | --- | --- | --- |
| *S. pierotii* M | 92,212,016 | 66,818,092 | 72.46% | 13,924,014,416 | 10,021,156,179 | 71.97% |
| *S. pierotii* F | 88,679,142 | 63,728,324 | 71.86% | 13,390,550,442 | 9,554,032,534 | 71.35% |
| *S. babylonica* M | 99,006,432 | 71,560,986 | 72.28% | 14,949,971,232 | 10,731,355,319 | 71.78% |
| *S. babylonica* F | 89,123,834 | 62,872,886 | 70.55% | 13,457,698,934 | 9,424,857,091 | 70.03% |
| *S. pseudolasiogyne* M | 91,882,824 | 66,138,908 | 71.98% | 13,874,306,424 | 9,918,622,034 | 71.49% |
| *S. pseudolasiogyne* F | 90,063,566 | 64,997,642 | 72.17% | 13,599,598,466 | 9,747,263,753 | 71.67% |

| Species | Aligned reads (#) | Coverage (X) | CP genome length (bp) |
| --- | --- | --- | --- |
| *S. pierotii* M | 1,704,882 | 1,666 | 155,688 |
| *S. pierotii* F | 1,232,836 | 1,204 | 155,688 |
| *S. babylonica* M | 3,404,884 | 3,327 | 155,695 |
| *S. babylonica* F | 1,128,480 | 1,103 | 155,695 |
| *S. pseudolasiogyne* M | 1,733,260 | 1,693 | 155,689 |
| *S. pseudolasiogyne* F | 1,391,013 | 1,359 | 155,689 |

**Supplementary Table 4.** Information on the assembled chloroplast genome. M: male, F: female.

**Supplementary Table 5.** Information on the assembled mitochondrial genome. M: male, F: female.

| Species | Aligned reads (#) | Coverage (X) | MT genome length (bp) |
| --- | --- | --- | --- |
| *S. pierotii* M | 1,611,052 | 345 | 705,081 |
| *S. pierotii* F | 1,369,825 | 293 | 705,081 |
| *S. babylonica* M | 2,393,927 | 512 | 705,179 |
| *S. babylonica* F | 1,288,026 | 276 | 705,179 |
| *S. pseudolasiogyne* M | 1,619,595 | 347 | 705,072 |
| *S. pseudolasiogyne* F | 1,815,064 | 389 | 705,072 |

**Supplementary Table 6.** Gene profile and organization of protein-coding genes in the mitochondrial genome of three *Salix* species.

| Gene | Length | Start Codon | Stop Codon | Direction |
| --- | --- | --- | --- | --- |
| *atp1* | 1524 | ATG | TAA | F |
| *atp4* | 597 | ATG | TAG | R |
| *atp6* | 714 | ATG | TAA | R |
| *atp8* | 474 | ATG | TAG | R |
| *atp9* | 225 | ATG | TAG | R |
| *ccmB* | 615 | ATG | TGA | R |
| *ccmC* | 753 | ATG | TGA | R |
| *ccmFc* | 1362 | ATG | TAG | R |
| *ccmFn* | 1725 | ATG | TGA | F |
| *cox1* | 1584 | ATG | TAA | F |
| *cox2* | 675 | ATG | TAA | R |
| *cox3* | 798 | ATG | TGA | F |
| *cob* | 1182 | ATG | TGA | R |
| *matR* | 1944 | ATG | TAG | F |
| *mttB* | 729 | TTG | TAG | F |
| *nad1* | 894 | ATG | TAA | F/R |
| *nad2* | 1461 | ATG | TAA | F/R |
| *nad3* | 357 | ATG | TAA | R |
| *nad4* | 1488 | ATG | TGA | R |
| *nad4L* | 303 | ATG | TAA | R |
| *nad5* | 2004 | ATG | TAA | F/R |
| *nad6* | 630 | ATG | TAG | F |
| *nad7* | 1185 | ATG | TAG | F |
| *nad9* | 573 | ATG | TAA | R |
| *rpl10* | 489 | ATG | TAA | F |
| *rpl16* | 411 | GTG | TAA | F |
| *rpl2* | 1029 | ATG | TAA | F |
| *rps1* | 690 | ATG | TAA | R |
| *rps12* | 378 | ATG | TGA | R |
| *rps14* | 141 | ATG | TAG | R |
| *rps3* | 1656 | ATG | TAA | F |
| *rps4* | 969 | ATG | TAA | R |
| *rps7* | 273 | ATG | TGA | F |
| *sdh4* | 396 | ATG | TAA | F |

**Supplementary Table 7.** Chloroplast and mitochondrial genomes downloaded from NCBI for phylogenetic tree analysis. **M: male, F: female.**

| No. | Species | GenBank accession number | |
| --- | --- | --- | --- |
|  |  | Chloroplast genome | Mitochondrial genome |
| 1 | *Salix pierotii* M | PQ842549 (155,688 bp) | PQ873106 (705,081 bp) |
| 2 | *Salix pierotii* F | PQ842550 (155,688 bp) | PQ873107 (705,081 bp) |
| 3 | *Salix babylonica* M | PQ842551 (155,695 bp) | PQ873108 (705,179 bp) |
| 4 | *Salix babylonica* F | PQ842552 (155,695 bp) | PQ873109 (705,179 bp) |
| 5 | *Salix pseudolasiogyne* M | PQ842553 (155,689 bp) | PQ873110 (705,072 bp) |
| 6 | *Salix pseudolasiogyne* F | PQ842554 (155,689 bp) | PQ873111 (705,072 bp) |
| 7 | *Salix wilsonii* | NC_053549 (155,026 bp) | NC_064688 (711,456 bp) |
| 8 | *Salix dunnii* | NC_058985 (155,647 bp) | NC_058734 (711,422 bp) |
| 9 | *Salix triandra* | NC_069592 (155,687 bp) | NC_069586 (735,196 bp) |
| 10 | *Salix suchowensis* | NC_026462 (155,214 bp) | NC_029317 (644,437 bp) |
| 11 | *Salix koriyanagi* | NC_044419 (155,548 bp) | NC_068760 (647,460 bp) |
| 12 | *Salix brachista* | NC_058984 (155,600 bp) | NC_058733 (608,983 bp) |
| 13 | *Salix integra* | NC_056253 (155,538 bp) | NC_068761 (606,136 bp) |
| 14 | *Salix purpurea* | NC_026722 (155,590 bp) | NC_029693 (598,970 bp) |
| 15 | *Populus davidiana* | NC_032717 (155,853 bp) | NC_035157 (779,361 bp) |

**Supplementary Table 8.** Best nucleotide substitution models for chloroplast and mitochondrial genomes in phylogenetic tree construction.

| CP CDS data set | Model | | | f(a) | | f(c) | | f(g) | f(t) | kappa | | titv | Ra | | Rb | Rc | | Rd | Re | Rf | | p-inv | gamma | |
| --- | --- | --- | --- | --- | --- | --- | --- | --- | --- | --- | --- | --- | --- | --- | --- | --- | --- | --- | --- | --- | --- | --- | --- | --- |
| AIC | GTR+I+G | | | 0.31 | | 0.19 | | 0.18 | 0.32 | 2.96 | | 1.38 | 0.8 | | 1.82 | 0.25 | | 0.63 | 1.8 | 1.00 | | 0.31 | 0.03 | |
| Model | | | -lnL* | | | | K | | | | AIC | | | Delta | | | Weight | | | | CumWeight | | |  |
| GTR+I+G | | | 100988.5 | | | | 37 | | | | 202051.1 | | | 0 | | | 0.66931 | | | | 0.66931 | | |  |
| TVM+I+G | | | 100988.4 | | | | 38 | | | | 202052.8 | | | 1.68566 | | | 0.288132 | | | | 0.957441 | | |  |
| TVM+I | | | 100993.1 | | | | 36 | | | | 202058.1 | | | 7.03346 | | | 0.019876 | | | | 0.977317 | | |  |
| TVM+G | | | 100993.8 | | | | 36 | | | | 202059.5 | | | 8.44974 | | | 0.00979 | | | | 0.987108 | | |  |
| GTR+I | | | 100992.9 | | | | 37 | | | | 202059.8 | | | 8.70606 | | | 0.008613 | | | | 0.99572 | | |  |
| MT CDS data set | | Model | | | f(a) | f(c) | | f(g) | f(t) | kappa | | titv | Ra | | Rb | Rc | | Rd | Re | Rf | | p-inv | gamma | |
| AIC | | GTR+I+G | | | 0.26 | 0.21 | | 0.21 | 0.32 | 1.27 | | 0.62 | 0.73 | | 0.97 | 0.20 | | 0.61 | 0.58 | 1.00 | | 0.92 | 0.03 | |
| Model | | | -lnL* | | | | K | | | | AIC | | | Delta | | | Weight | | | | CumWeight | | |  |
| GTR+I+G | | | 38383.7 | | | | 38 | | | | 76843.4 | | | 0 | | | 0.407634 | | | | 0.407634 | | |  |
| TVM+I+G | | | 38385.0 | | | | 37 | | | | 76843.9 | | | 0.50318 | | | 0.316961 | | | | 0.724594 | | |  |
| TVM1+I+G | | | 38386.8 | | | | 36 | | | | 76845.6 | | | 2.1497 | | | 0.139145 | | | | 0.86374 | | |  |
| TPM1uf+I | | | 38388.0 | | | | 35 | | | | 76846.0 | | | 2.61732 | | | 0.110135 | | | | 0.973875 | | |  |
| TIM2+I+G | | | 38389.4 | | | | 36 | | | | 76850.8 | | | 7.41734 | | | 0.009991 | | | | 0.983866 | | |  |

**Supplementary Table 9.** Gene composition of the chloroplast genome of *Salix pierotii*, *S. babylonica*, and *S. pseudolasiogyne*.

| Group of genes | Name of genes |
| --- | --- |
| Photosystem Ⅰ | *psaA*, *B*, *C*, *I*, *J*, *pafI*^2)^, *pafII* |
| Photosystem Ⅱ | *psbA*, *B*, *C*, *D*, *E*, *F*, *H*, *I*, *J*, *K*, *L*, *M*, *T*, *Z*, *pbf1* |
| Cytochrome b6/f | *petA*, *B*^1)^, *D*^1)^, *G*, *L*, *N* |
| ATP synthase | *atpA*, *B*, *E*, *F*^1)^, *H*, *I* |
| Rubisco | *rbcL* |
| NADH oxidoreductase | *ndhA*^1)^, *B*^1),3)^, *C*, *D*, *E*, *F*, *G*, *H*, *I*, *J*, *K* |
| Large subunit ribosomal proteins | *rpl2*^1),3)^, *14*, *16*^1)^, *20*, *22*, *23*^3)^, *33*, *36* |
| Small subunit ribosomal proteins | *rps2*, *3*, *4*, *7*^3)^, *8*, *11*, *12*^2),3),4)^, *14*, *15*, *18*, *19*^3)^ |
| RNA polymerase | *rpoA*, *B*, *C1*^1)^, *C2* |
| Unknown function protein coding gene | *ycf1*^3)^, *ycf2*^3)^, *ycf15*^3)^ |
| Other genes | *accD*, *ccsA*, *cemA*, *clpP1*^2)^, *matK* |
| Ribosomal RNAs | *rrn4.5*^3)^, *rrn5*^3)^, *rrn16*^3)^, *rrn23*^3)^ |
| Transfer RNAs | *trnA*-*UGC*^1),3)^, *trnC*-*GCA*, *trnD*-*GUC*, *trnE*-*UUC*, *trnF*-*GAA*, *trnfM*-*CAU*, *trnG*-*GCC*^1)^, *trnG*-*UCC*, *trnH*-*GUG*, *trnl*-*CAU*^3)^, |
|  | *trnl*-*GAU*^1),3)^, *trnK*-*UUU*^1)^, *trnL*-*CAA*^3)^, *trnL*-*UAA*^1)^, *trnL*-*UAG*, *trnM*-*CAU*, *trnN*-*GUU*^3)^, *trnP*-*UGG*, *trnQ*-*UUG*, *trnR*-*ACG*^3)^, *trnR*-*UCU*, *trnS*-*GCU*, *trnS*-*GGA*, *trnS*-*UGA*, *trnT*-*GGU*, *trnT*-*UGU*, *trnV*-*GAC*^3)^, *trnV*-*UAC*^1)^, *trnW*-*CCA*, *trnY*-*GUA* |

^1)^ Gene containing a single intron, ^2)^ Gene containing two introns, ^3)^ Two gene copies in IRs, ^4)^ Trans-splicing gene

**Supplementary Table 10.** Intron and exon in three *Salix* chloroplast genome.

| *S. babylonica* | Gene | Region | Exon I | Intron I | Exon II | Intron II | Exon III |
| --- | --- | --- | --- | --- | --- | --- | --- |
| 1 | *trnK-UUU* | LSC | 37 | 2530 (2551)^1,2^ | 29 |  |  |
| 2 | *trnG-UCC* | LSC | 23 | 703 (702)^1,2^ | 48 |  |  |
| 3 | *atpF* | LSC | 144 | 732 (730)^1,2^ | 411 |  |  |
| 4 | *rpoC1* | LSC | 453 | 782 | 1617 |  |  |
| 5 | *pafI* | LSC | 126 | 724 | 228 | 729 | 153 |
| 6 | *trnL-UAA* | LSC | 35 | 597 (576)^1,2^ | 50 |  |  |
| 7 | *trnV-UAC* | LSC | 39 | 607 | 37 |  |  |
| 8 | *rps12* | LSC | 114 |  | 231 |  | 27 |
| 9 | *clpP1* | LSC | 71 | 826 | 292 | 616 (596)^1,2^ | 228 |
| 10 | *petB* | LSC | 6 | 820 | 642 |  |  |
| 11 | *petD* | LSC | 9 | 785 (783)^1,2^ | 489 |  |  |
| 12 | *rpl16* | LSC | 9 | 1122 (1130)^1^ (1131)^2^ | 399 |  |  |
| 13 | *rpl2* | IR | 396 | 668 | 435 |  |  |
| 14 | *ndhB* | IR | 777 | 682 | 756 |  |  |
| 15 | *trnI-GAU* | IR | 42 | 942 | 35 |  |  |
| 16 | *trnA-UGC* | IR | 38 | 801 | 35 |  |  |
| 17 | *ndhA* | SSC | 552 | 1087 (1086)^1,2^ | 546 |  |  |

^1^ Parentheses indicate *S. pierotii*, ^2^ Parentheses indicate *S. pesuodolasiogyne*

**Supplementary Table 11.** Gene composition of the mitochondrial genome of *Salix pierotii*, *S. babylonica*, and *S. pseudolasiogyne*.

| Group of genes | Name of genes |
| --- | --- |
| NADH dehydrogenase | *nad1**, *nad2**, *nad3*, *nad4**, *nad4L*, *nad5**, *nad6, nad7**, *nad9* |
| ATP synthase | *atp1*, *atp4*, *atp6*, *atp8*, *atp9* |
| Cytochrome c biogenesis | *ccmB*, *ccmC*, *ccmFc**, *ccmFn* |
| Cytochrome c oxidase | *cox1*, *cox2*, *cox3* |
| Maturases | *matR* |
| Ubiquinol cytochrome c reductase | *cob* |
| Ribosomal proteins (LSU) | *rpl10*, *rpl16*, *rpl2** |
| Ribosomal proteins (SSU) | *rps1*, *rps12*, *rps14*, *rps3**, *rps4*, *rps7* |
| Transport membrane protein | *mttB* |
| Succinate Dehydrogenase | *sdh4* |
| Ribosomal RNAs | *rrn5*, *rrnL*, *rrnS* |
| Transfer RNAs | *trnC*-*GCA*, *trnD*-*GUC*, *trnE*-*UUC*, *trnF*-*GAA*, *trnG*-*GCC*, *trnH*-*AUG*, *trnH*-*GUG*, *trnK*-*UUU*, *trnM*-*CAU* (×3), *trnN*-*GUU*, *trnP*-*UGG* (×2), *trnQ*-*UUG*, *trnS*-*GCU*, *trnS*-*GGA*, *trnS*-*UGU*, *trnV*-*GAC*, *trnW*-*CCA*, *trnY*-*GUA*, *trnfM*-*CAU* |

* Labeled intron containing genes

**Supplementary Table 14.** Genes associated with complete transferred DNA fragments from chloroplasts to mitochondria. Detailed information can be found in Supplementary Table 15.

| Group | Complete Transferred Genes |
| --- | --- |
| I (other sections) | *atpE* |
| II (sect. *Wilsonia*) | None |
| III (sect. *Salix*) | *psaA*, *psbC*, *D*, *H*, *psbf1*, *petB*, *atpE* |
